# Clinical prediction models to diagnose neonatal sepsis in low-income and middle-income countries: a scoping review

**DOI:** 10.1136/bmjgh-2024-017582

**Published:** 2025-04-09

**Authors:** Samuel R Neal, Sarah S Sturrock, David Musorowegomo, Hannah Gannon, Michele Zaman, Mario Cortina-Borja, Kirsty Le Doare, Michelle Heys, Gwendoline Chimhini, Felicity Fitzgerald

**Affiliations:** 1 UCL GOS Institute of Child Health, London, UK; 2 The University of Edinburgh College of Medicine and Veterinary Medicine, Edinburgh, UK; 3 St George’s University of London, London, UK; 4 University of Zimbabwe Faculty of Medicine and Health Sciences, Harare, Zimbabwe; 5 Queen’s University School of Medicine, Kingston, Ontario, Canada; 6 Imperial College London, London, UK

**Keywords:** Global Health, Mathematical modelling, Decision Making, Paediatrics, Systematic review

## Abstract

**Introduction:**

Neonatal sepsis causes significant morbidity and mortality worldwide but is difficult to diagnose clinically. Clinical prediction models (CPMs) could improve diagnostic accuracy, facilitating earlier treatment for cases and avoiding antibiotic overuse. Neonates in low-income and middle-income countries (LMICs) are disproportionately affected by sepsis, yet no review has comprehensively synthesised evidence for CPMs validated in this setting.

**Methods:**

We performed a scoping review of CPMs to diagnose neonatal sepsis using Ovid MEDLINE, Ovid Embase, Scopus, Web of Science, Global Index Medicus and the Cochrane Library. The most recent searches were performed on 16 June 2024. We included studies published in English or Spanish that validated a new or existing CPM for neonatal sepsis in any healthcare setting in an LMIC. Studies were excluded if they validated a prognostic model or where data for neonates could not be separated from a larger paediatric population. Studies were selected by two independent reviewers and summarised by narrative synthesis.

**Results:**

From 4598 unique records, we included 82 studies validating 44 distinct models in 24 252 neonates. Most studies were set in neonatal intensive or special care units (n=64, 78%) in middle-income countries (n=81, 99%) and included neonates already suspected of sepsis (n=58, 71%). Only four studies (5%) were set in the WHO African region, and only one study included data from a low-income country. Two-thirds of CPMs (n=30) required laboratory parameters, and three-quarters (n=34) were only validated in one study.

**Conclusion:**

Our review highlights several literature gaps, particularly a paucity of studies validating models in the lowest-income countries where neonatal sepsis is most prevalent, and models for the undifferentiated neonatal population that do not rely on laboratory tests. Furthermore, heterogeneity in study populations, definitions of sepsis and reporting of models inhibits meaningful comparison between studies and may hinder progress towards useful diagnostic tools.

**WHAT IS ALREADY KNOWN ON THIS TOPIC**

- Multiple clinical prediction models exist to diagnose neonatal sepsis, and these have been validated in a range of countries across different resource and income contexts.

**WHAT THIS STUDY ADDS**

- Prior to our review, there was no comprehensive synthesis of models validated in low-income and middle-income countries: those countries with the highest incidence of neonatal sepsis and least access to specialist neonatal care.

**HOW THIS STUDY MIGHT AFFECT RESEARCH, PRACTICE OR POLICY**

- There is a large and increasing number of studies validating clinical prediction models to diagnose neonatal sepsis in low-income and middle-income countries, which could be harnessed to improve diagnostic accuracy, reduce time to diagnosis and rationalise antibiotic use.
- Future research should address the literature gaps identified in our review, including a paucity of studies validating models in low-income countries and the WHO African region, and models designed to assess risk of sepsis in an undifferentiated neonatal population without pre-existing suspicion of sepsis.
- Greater adherence to standardised reporting guidelines and agreement on a consensus definition of neonatal sepsis would improve the quality and comparability of future studies.

## Introduction

 Neonatal sepsis is a clinical syndrome caused by severe systemic infection in the first month of life.^1^ It is a leading cause of global neonatal mortality and disproportionately affects neonates in low-income and middle-income countries (LMICs).^2^

Conventionally, neonatal sepsis is divided into early-onset sepsis (EOS) usually due to vertical transmission of pathogens from the maternal genitourinary tract and occurring in the first 48–72 hours of life and late-onset sepsis (LOS) usually due to pathogens acquired from the home or hospital environment and occurring after 48–72 hours of life.^3^ An exception to this typology is infection caused by group B streptococcus (GBS), where EOS is often considered to occur up to the seventh day of life.^3^ However, there is increasing recognition that this conventional classification of neonatal sepsis is misplaced in LMICs where neonates may be exposed from birth to organisms traditionally associated with LOS due to high rates of healthcare-associated infections.^4^

Neonatal sepsis is difficult to diagnose clinically due to non-specific signs and symptoms. Identifying a pathogenic organism from a normally sterile site (eg, blood or cerebrospinal fluid (CSF)) remains the gold standard method for diagnosis.^3^ Nevertheless, *‘*clinical’ or ‘culture-negative’ sepsis—where a sterile culture is obtained from a neonate with signs and symptoms of sepsis—is a recognised entity.^5^ When sepsis is suspected, a fine balance must be struck between the risk of failing to treat a true invasive infection and the risks of unnecessary antimicrobial use, which can contribute to antimicrobial resistance,^4^ and adverse neonatal outcomes.^6^ Additionally, guidelines recommend starting antimicrobial therapy as soon as possible (the UK specifies within 1 hour of suspecting sepsis^7^) to maximise the chance of survival, leaving little time for clinicians to confirm a diagnosis.

Clinical prediction models (CPMs) aim to estimate the individual probability of a diagnosis or prognostic outcome.^8^ Once the research question and prediction problem have been defined, there are several steps to develop a valid CPM. Most salient to this review are variable selection, model specification, model estimation and evaluation of model performance (eg, discrimination and calibration) through apparent validation, internal validation and external validation. We define these terms in box 1.

Box 1 **Definitions of key terms in clinical prediction model development and validation**

**Model development**

- Variable selection: the process of choosing which subset of predictors to include in the model. Variable selection may be based on expert opinion, literature review and/or statistical procedures such as stepwise selection, which are based on goodness-of-fit criteria.
- Model specification: the process of choosing the functional systematic form of the model. This includes selection of main effects (variable selection) and how to handle model assumptions, including additivity and non-linearity, with particular concern to avoid overfitting.
- Model estimation: the process of estimating model parameters. Regression coefficients are usually estimated using least squares for linear regression and maximum likelihood for logistic regression. Modern estimation methods include uniform shrinkage (eg, heuristic or bootstrapping techniques) and penalisation (eg, ridge regression or lasso).

**Model validation**

- Validation: the process of evaluating model performance, including overall performance, discrimination and calibration.
- Apparent validation: assessment of model performance directly in the sample used for model development (the derivation cohort). This can be considered a limited form of internal validation.
- Internal validation: assessment of model performance in the sample used for model development using techniques such as split-sample validation, cross-validation or bootstrapping.
- External validation: assessment of model performance in a sample other than that used for model development. This includes validation in a more recent sample (temporal validation) and in samples from different sites (geographic validation).
- Discrimination: the ability of a model to differentiate between those with and without the disease or outcome. Commonly assessed by calculating sensitivity, specificity and area under the receiver operating characteristic curve.
- Calibration: the agreement between model predictions and the observed outcomes. May be assessed using the calibration intercept (‘calibration-in-the-large’), calibration slope (‘weak calibration’) and calibration plot (‘moderate calibration’).

These definitions are adapted from Steyerberg.^8^ We direct the reader to this excellent resource for a more detailed review of these topics.

CPMs are available to diagnose neonatal sepsis in both high-income countries and LMICs based on various clinical features, risk factors and/or laboratory tests. These CPMs are designed to improve diagnostic accuracy and reduce time to diagnosis to promote timely, judicious antibiotic prescribing. The benefits of early recognition and treatment could be significant for neonates in low-resource settings where specialist care is limited.^9^ CPMs may also be valuable diagnostic tools where laboratory diagnostics are costly or time-consuming. Furthermore, reducing the growing threat of antimicrobial resistance is crucial where access to sufficiently broad-spectrum antimicrobials may be unaffordable for patients.^10^

While several existing reviews examine CPMs to diagnose neonatal sepsis,^11^,^14^ there has been no comprehensive synthesis of models validated in LMICs. Therefore, the aim of this scoping review was to map the literature of CPMs to diagnose neonatal sepsis in LMICs.

Specific objectives were:

1. To provide an overview of existing CPMs to diagnose neonatal sepsis in LMICs
2. To provide an overview of the characteristics and methodology of studies that develop and/or validate CPMs to diagnose neonatal sepsis in LMICs
3. To compare the performance of CPMs using different approaches to risk stratification or different target populations
4. To identify unanswered research questions surrounding CPMs to diagnose neonatal sepsis in LMICs, which may guide future primary research or systematic reviews

## Methods

We conducted this review according to an a priori published protocol,^15^ developed with reference to the scoping review guidelines provided by the Joanna Briggs Institute.^16^ We report methods and results in accordance with the Preferred Reporting Items for Systematic reviews and Meta-Analyses extension for Scoping Reviews (see online supplemental appendix).^17^

We conducted a scoping review, instead of a systematic review, as we anticipated a broad, heterogeneous body of literature describing diverse CPMs and settings in which they had been studied.^18^ Within the scoping review framework, we aimed to provide an overview of the quantity and types of evidence for CPMs for neonatal sepsis in LMICs, instead of definitively recommending the use of a single model.

### Search strategy

Eligibility criteria are shown in table 1. After reviewing the extent and breadth of the literature from our initial searches, we narrowed the scope of our original protocol to focus specifically on studies that validate a CPM to diagnose neonatal sepsis in a LMIC, as defined by the World Bank in 2020.^19^

**Table 1:** Eligibility criteria

Population	Neonates aged ≤30 days of life, or hospitalised in a neonatal unit, being evaluated for neonatal sepsis. The definition of neonatal sepsis was left to the discretion of individual studies. Studies examining a range of patient ages were included, provided that sufficient data were available to examine model performance in neonates specifically.
Concept	Studies that develop or validate a CPM to diagnose neonatal sepsis in a LMIC, as defined by the World Bank in 2020. Studies were considered to have validated a CPM if they reported any of: *C*-statistic denoting AUC, sensitivity, specificity, predictive values, likelihood ratios or changes in clinical practice or outcomes through apparent, internal or external validation. Studies evaluating prognostic models (eg, to predict mortality or morbidity) were excluded.
Context	Studies from any healthcare setting, including neonatal units, emergency departments, outpatient settings or community settings. Studies were only included if the model they refer to has been internally and/or externally validated in a LMIC, regardless of the country where the model was initially developed.
Type of studies	Randomised and quasi-randomised controlled trials, cohort studies, cross-sectional studies, case-control studies and clinical guidelines. Also, letters, comments and conference proceedings (providing sufficient details were provided for data extraction). Systematic reviews, meta-analyses and editorials were excluded, but were used to identify relevant primary literature. No time period restrictions, but only studies published in English or Spanish were considered, to reflect the languages spoken by the review team.

AUC, area under the receiver operating characteristic curve; CPM, clinical prediction model; LMIC, low-income and middle-income country.

We searched six electronic databases from their inception: Ovid MEDLINE, Ovid Embase, Scopus, Web of Science Core Collection, Global Index Medicus and the Cochrane Library. Searches were initially performed on 20 December 2019 and updated on 5 September 2022 and 16 June 2024. Search terms were chosen to capture the three domains of the research question (‘neonate’, ‘sepsis’ and ‘clinical prediction model’) through collaboration with a child health specialist librarian. The search strategy was developed for Ovid MEDLINE and adapted for each database (see online supplemental appendix). Additional studies were identified by citation analysis and by hand searching the reference lists of included studies and relevant reviews.

We used a broad working definition of ‘clinical prediction model’ since there is no universal definition. We defined a CPM as any tool with at least three predictors (covariates) where the intention was to estimate an individual’s probability of neonatal sepsis being present and/or to classify them as ‘septic’ or ‘non-septic’.^20^ For this review, we extended the definition of CPM to include decision rules or scoring systems developed without modelling in the strict sense, for example, tools derived by assigning scores to predictors based on their perceived importance from literature review or expert opinion, or simply by scoring a predictor as present or absent. Tools could be presented in any form, including a regression model formula, scoring system or chart, nomogram or decision tree.

### Record screening

We imported identified records into EndNote 21 for deduplication.^21^ Unique records were then uploaded to the Rayyan application for screening by two independent reviewers (DM, HG, MZ, SRN or SS).^22^ Titles and abstracts were first examined against the eligibility criteria to determine if each record was potentially eligible for inclusion. Next, full texts of potentially eligible studies were obtained and reviewed to confirm eligibility. Authors were contacted to request full texts where these could not be found online. Conflicts were resolved by discussion among the review team, with a third reviewer acting as a tiebreaker if needed. Percentage agreement was calculated by dividing the number of studies with independent agreement from all reviewers by the total number of records screened.

### Data extraction and synthesis

Data extraction was performed independently by two reviewers for the initial searches (SRN and SS) and by one reviewer for each updated search (SRN or SS). We extracted data on study, participant and model characteristics, and model performance using a pre-piloted data extraction form (see online supplemental appendix). Data items were chosen based on the Transparent Reporting of a multivariable prediction model for Individual Prognosis Or Diagnosis (TRIPOD) statement.^23^ We summarised results by narrative synthesis. Data for quantitative outcomes were not pooled in a meta-analysis as this is beyond the scoping review methodology. Where multiple variations of a model were presented in the same study (eg, different combinations of predictors presented during model specification), or model performance was presented at multiple classification thresholds, we only included data for the ‘optimal’ or ‘final’ model at a single classification threshold. We extracted performance metrics in the following order of priority: external validation, internal validation and apparent validation. If a study updated or modified an existing model, this new model was included separately if it was deemed to be materially different from the original.

We extracted definitions of terms identifying the populations studied where these were given. Of note, there is no international definition of the distinction between neonatal intensive and special care. Units defining themselves as special care units usually provide care for later preterm babies or those requiring a lower level of support than intensive care units (which can usually provide organ support such as mechanical ventilation).^24^

### Patient and public involvement

Neither patients nor the public were involved in the design or reporting of this study.

### Ethics statement

Ethical approval was not required as this study used only publicly available data.

## Results

### Searches and included studies

**Figure 1:**
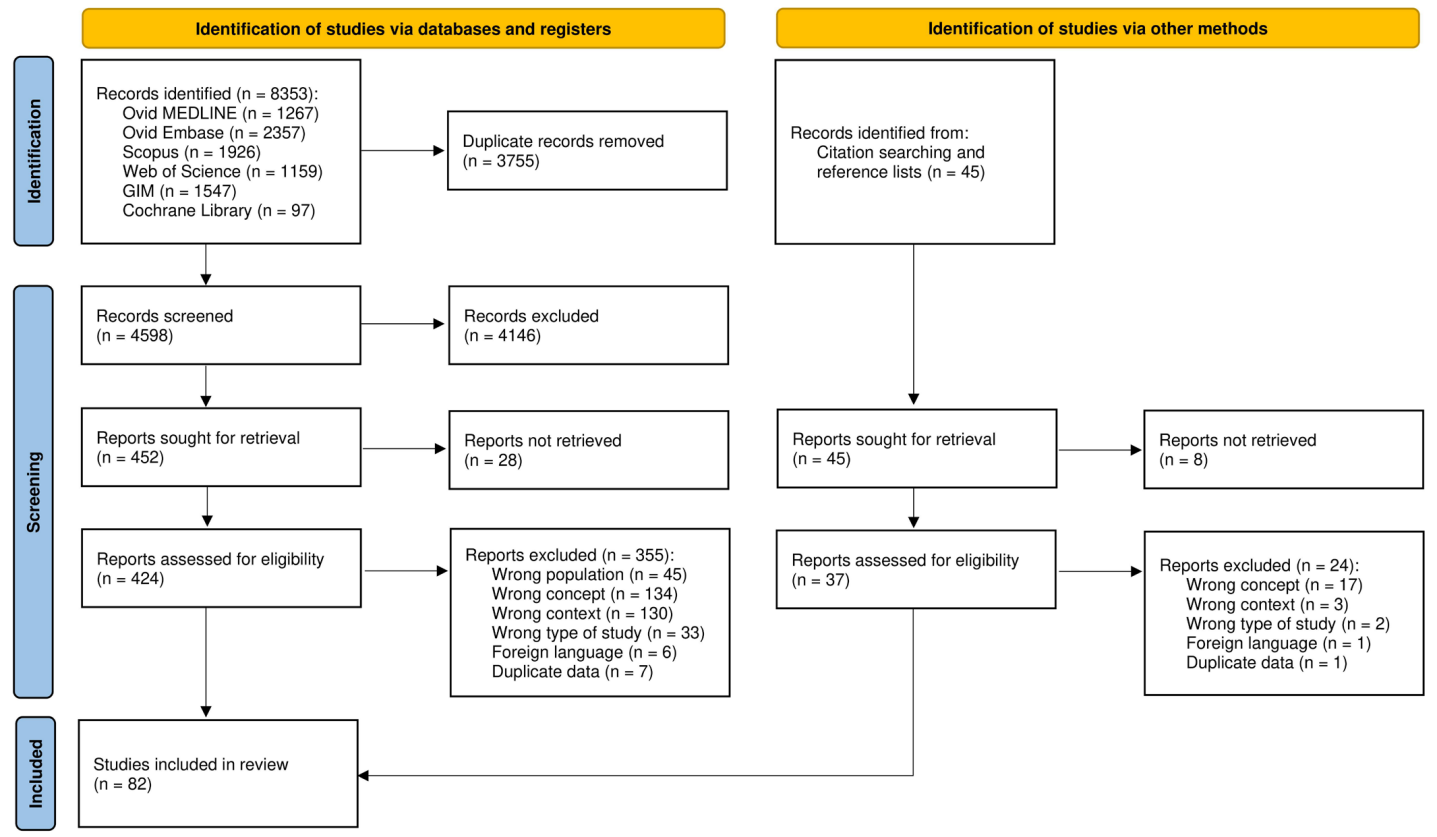
Preferred Reporting Items for Systematic Reviews and Meta-Analyses (PRISMA) flowchart of study selection. GIM, Global Index Medicus. Adapted from Page *et al*.^132^

**Figure 2:**
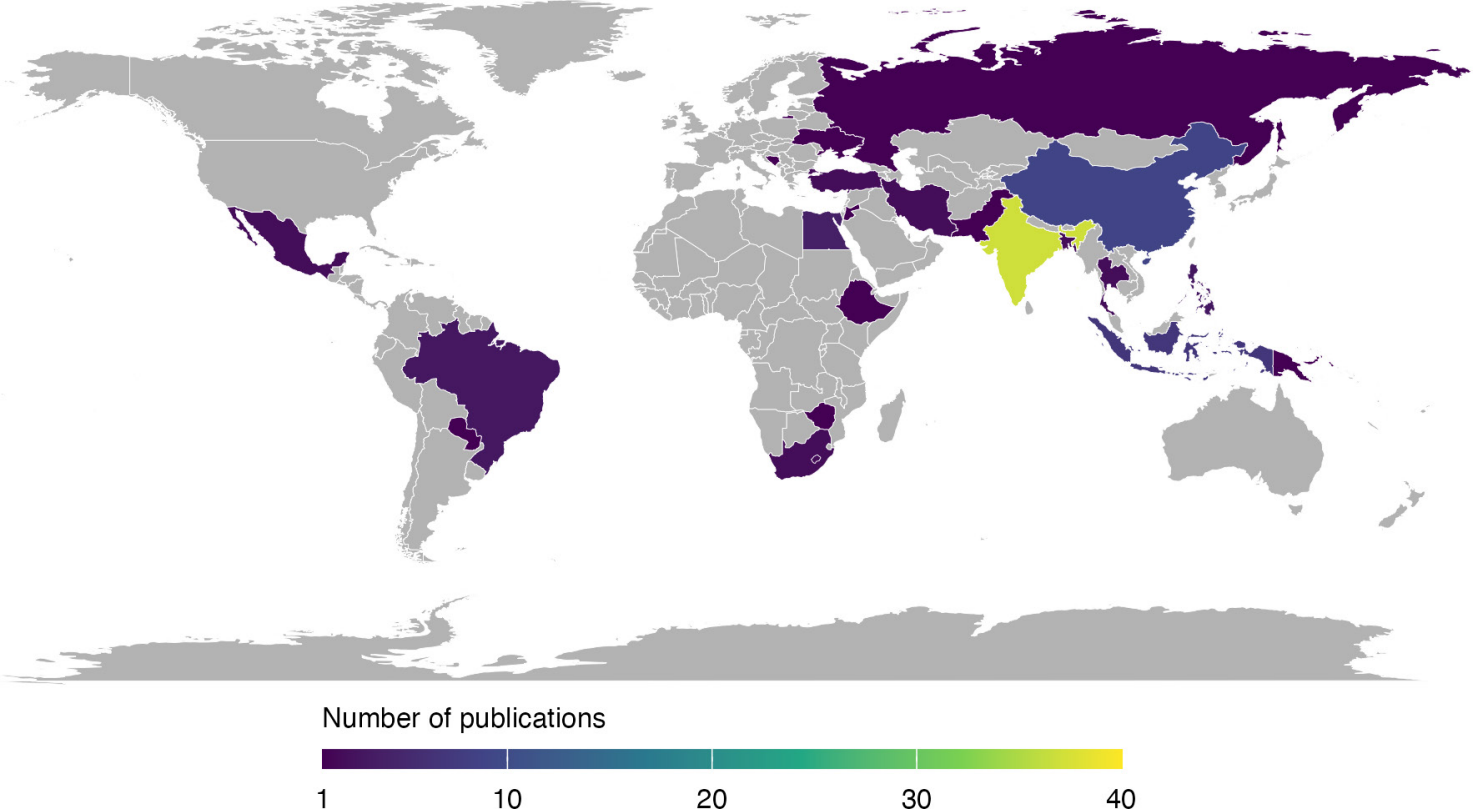
Choropleth map of the number of included studies by country.

Searches identified 4598 unique records (figure 1). From these, 82 studies published between August 2003 and May 2024 were included^25^,^106^ and are summarised in tables2  3 and online supplemental tables 1 and 2. There was 98% inter-reviewer agreement on study inclusion. The number of published studies validating a CPM to diagnose neonatal sepsis in LMICs has increased rapidly in recent years (online supplemental figure 1). Studies were conducted in 22 individual countries (figure 2 and online supplemental tables 3 and 4), with the greatest number of studies conducted in the WHO South-East Asian Region (n*=*48, 59%), particularly in India (n*=*37, 45%). The fewest studies were conducted in the WHO African Region (n*=*4, 5%). Regarding economic status, 51 studies were conducted exclusively in lower middle-income countries (62%) and 30 exclusively in upper middle-income countries (37%) (online supplemental table 5). One study pooled data from both low-income and lower middle-income countries.^101^ Most studies were set in intensive care or special care admission units (n*=*64, 78%). The remainder included all live births at study sites (n*=*12, 15%), neonates presenting to emergency care services (n*=*3, 4%), all hospitalised neonates (n=1, 1%) or the setting was unclear (n*=*2, 2%).

**Table 2:** Summary of included studies and model performance

Model	Study, country
Abiramalatha	Abiramalatha *et al*^25^ 2016, India
Ahire	Ahire *et al*^27^ 2022, India
Ahmed	Ahmed *et al*^28^ 2005, Pakistan
American Academy of Pediatrics (AAP)	Beandda *et al*^32^ 2019, the Philippines
Bekhof	Lloyd *et al*^65^ 2022, South Africa
Boston protocol	Bulbul *et al*^35^ 2020, Turkey
Celik	Celik *et al*^36^ 2013, Turkey
Fitriana	Fitriana *et al*^43^ 2023, Indonesia
He	He *et al*^49^ 2017, China
Helguera-Repetto	Helguera-Repetto *et al*^50^ 2020, Mexico
Hematological Scoring System (HSS)	Amir *et al*^30^ 2023, India
	Annam *et al*^31^ 2015, India
	Bhalodia *et al*^33^ 2017, India
	Debroy *et al*^39^ 2016, India
	Derbala *et al*^40^ 2017, Egypt
	Dutta *et al*^41^ 2016, India
	El-Said *et al*^42^ 2024, Egypt
	Gupta *et al*^45^ 2011, India
	Ibrahim *et al*^55^ 2023, Egypt
	Khair *et al*^61^ 2012, India
	Krishnamurthy *et al*^62^ 2017, India
	Liestiadi *et al*^64^ 2017, Indonesia
	Makkar *et al*^68^ 2013, India
	Malini *et al*^69^ 2016, India
	Manvitha *et al*^70^ 2023, India
	Meirina *et al*^72^ 2015, Indonesia
	Mishra *et al*^73^ 2019, India
	Nair *et al*^77^ 2020, India
	Narasimha *et al*^78^ 2011, India
	Padhy *et al*^82^ 2023, India
	Pramana *et al*^85^ 2016, Indonesia
	Shah *et al*^91^ 2019, India
	Shukla *et al*^94^ 2023, India
	de Souza^38^ 2015, Brazil
HSS (cord blood)	Himasree *et al*^51^ 2024, India
	Shukla *et al*^94^ 2023, India
HSS (+) CRP	Amir *et al*^30^ 2023, India
	Khair *et al*^61^ 2012, India
	Nabi *et al*^76^ 2019, Bangladesh
HSS (+) PCR	Godoy Torales *et al*^44^ 2020, Paraguay
HSS (+) CRP, micro-ESR	Amir *et al*^30^ 2023, India
HSS (+) nucleated RBCs (-) I:M ratio(‘Sepscore’)	Padhy *et al*^82^ 2023, India
	Sharma *et al*^92^ 2024, India
HSS (+) nucleated RBCs (-) I:M ratio, immature PMN count(‘modified HSS’)	Ibrahim *et al*^55^ 2023, Egypt
	Krishnamurthy *et al*^62^ 2017, India
HSS (+) CRP, CD64 (-) PMN changes	Mohamed *et al*^74^ 2012, Egypt
HSS (+) cord CRP, 48-hour CRP (-) I:T ratio	Varughese *et al*^99^ 2019, India
HSS (+) nucleated RBCs (-) I:M ratio, PMN changes	Chitra *et al*^37^ 2022, Indonesia
HSS (+) nucleated RBCs, MPV, PDW (-) I:M ratio, PMN changes	Chitra *et al*^37^ 2022, Indonesia
HSS (+) CRP, partial blood culture result(‘ANVISA handbook protocol’)	Pinto *et al*^83^ 2013, Brazil
Hu	Hu *et al*^52^ 2021, China
Huang	Huang *et al*^53^ 2020, China
	Shuai *et al*^93^ 2022, China
Husada EOS	Husada *et al*^54^ 2010, Thailand
Husada LOS	Husada *et al*^54^ 2010, Thailand
Iqbal	Iqbal *et al*^57^ 2024, India
Istanbul protocol	Bulbul *et al*^35^ 2020, Turkey
Kaiser Permanente EOS Calculator	Al-Lawama *et al*^29^ 2019, Jordan
	Beandda *et al*^32^ 2019, the Philippines
	He *et al*^48^ 2020, China
	Ikhsaniatun *et al*^56^ 2022, Indonesia
Kar	Kar *et al*^60^ 2010, India
Matsushita	Matsushita *et al*^71^ 2022, Brazil
Mondal	Mondal *et al*^75^ 2012, India
Neal	Neal *et al*^79^ 2023, Zimbabwe
NeoHoP	Lloyd *et al*^66^ 2023, South Africa
Nguyen	Nguyen *et al*^80^ 2023, China
NOSEP-1	Lloyd *et al*^65^ 2022, South Africa
	Reyna-Figueroa *et al*^88^ 2005, Mexico
NOSEP-NEW1	Lloyd *et al*^65^ 2022, South Africa
Okascharoen	Okascharoen *et al*^81^ 2005, Thailand
	Raguindin *et al*^87^ 2014, the Philippines
Perinatal Infection Risk Score	Hassan *et al*^47^ 2016, India
	Sriram *et al*^96^ 2011, India
Philadelphia protocol	Bulbul *et al*^35^ 2020, Turkey
Pokhylko	Pokhylko *et al*^84^ 2020, Ukraine
PROM-Scoring	Afjeiee *et al*^26^ 2008, Iran
Pukhtinskaya	Pukhtinskaya and Estrin^86^ 2021, Russia
Rochester protocol	Bulbul *et al*^35^ 2020, Turkey
	Zarkesh *et al*^105^ 2011, Iran
Rosenberg	Lloyd *et al*^65^ 2022, South Africa
	Rosenberg *et al*^89^ 2010, Bangladesh
Selimovic	Selimovic *et al*^90^ 2010, Bosnia and Herzegovina
Septic screen	Buch *et al*^34^ 2011, India
	Gupta *et al*^46^ 2022, India
	Hassan *et al*^47^ 2016, India
	Jadhav *et al*^58^ 2013, India
	Kudawla *et al*^63^ 2008, India
	Mahale *et al*^67^ 2010, India
	Sriram^96^ 2011, India
	Swarnkar *et al*^97^ 2012, India
	Thermiany *et al*^98^ 2008, Indonesia
	Vinay *et al*^100^ 2015, India
	Yadav *et al*^103^ 2023, India
Shuai	Shuai *et al*^93^ 2022, China
Singh	Singh *et al*^95^ 2003, India
	Kudawla *et al*^63^ 2008, India
	Rosenberg *et al*^89^ 2010, Bangladesh
	Lloyd *et al*^65^ 2022, South Africa
Singh (+) septic screen	Kudawla *et al*^63^ 2008, India
STOPS tool	James *et al*^59^ 2021, India
Weber	Weber *et al*^101^ 2003, Ethiopia, the Gambia, Papua New Guinea and the Philippines
Wu	Wu *et al*^102^ 2024, China
Yadav	Yadav *et al*^103^ 2023, India
Yin	Yin *et al*^104^ 2022, China
Zhang	Zhang *et al*^106^ 2023, China

Full table available in online supplemental appendix.

ANVISA, Agência Nacional de Vigilância Sanitária; CD, cluster of differentiation; CRP, C-reactive protein; EOS, early-onset sepsis; ESR, erythrocyte sedimentation rate; LOS, late-onset sepsis; MPV, mean platelet volume; PCR, PCR chain reaction; PDW, platelet distribution width; PMN, polymorphonuclear neutrophil; PROM, premature rupture of membranes; I:T ratio, immature to total neutrophil ratio; I:M ratio, immature to mature neutrophil ratio; RBC, red blood cell.

**Table 3:** Summary of model characteristics

Model (derivation study)	Country of derivation cohort	Outcome	Modelling methods	Predictors*
Abiramalatha (Abiramalatha *et al*^25^ 2016)	India	All sepsis	Scoring system	L
Ahire (Ahire *et al*^27^ 2022)	India	All sepsis	Scoring system	L
Ahmed (Ahmed *et al*^28^ 2005)	Pakistan	All sepsis	Scoring system	L
American Academy of Pediatrics (AAP) (Puopolo *et al*^114^ 2018)	USA	EOS	Criteria-based without specific scoring	C, R
Bekhof (Bekhof *et al*^109^ 2013)	The Netherlands	LOS	Nomogram from logistic regression	C, R
Boston protocol (Baskin *et al*^108^ 1992)	USA	Serious bacterial infection	Criteria-based without specific scoring	C, R, L
Celik (Celik *et al*^36^ 2013)	Turkey	All sepsis	Markov state models	L
Fitriana (Fitriana *et al*^43^ 2023)	Indonesia	EOS	Logistic regression	R
Hematological Scoring System (Rodwell *et al*^115^ 1988)	Australia	All sepsis	Scoring system	L
He (He *et al*^49^ 2017)	China	EOS	Logistic regression	L
Helguera-Repetto (Helguera-Repetto *et al*^50^ 2020)	Mexico	All sepsis	Neural network	C, R, L
Hu (Hu *et al*^52^ 2021)	China	EOS	Logistic regression	C, L
Huang (Huang *et al*^53^ 2020)	China	LOS	Nomogram from logistic regression	R, L
Husada EOS (Husada *et al*^54^ 2010)	Thailand	EOS	Logistic regression	C, R, L
Husada LOS (Husada *et al*^54^ 2010)	Thailand	LOS	Logistic regression	C, L
Iqbal (Iqbal *et al*^57^ 2024)	India	All sepsis	Supervised machine learning	C, R, L
Istanbul protocol (Bulbul *et al*^35^ 2020)	Turkey	Serious bacterial infection	Criteria-based without specific scoring	C, R, L
Kaiser Permanente EOS Calculator (Kuzniewicz *et al*^110^ 2017)	USA	EOS	Bayesian logistic regression and recursive partitioning	C, R
Kar (Kar *et al*^60^ 2010)	India	LOS	Scoring system	C
Matsushita (Matsushita *et al*^71^ 2022)	Brazil	All sepsis	Supervised machine learning	L
Mondal (Mondal *et al*^75^ 2012)	India	All sepsis	Scoring system	L
Neal (Neal *et al*^79^ 2023)	Zimbabwe	EOS	Logistic regression	C, R
NeoHoP (Lloyd *et al*^66^ 2023)	South Africa	LOS (HAI)	Logistic regression	C, R, L
Nguyen (Nguyen *et al*^80^ 2023)	China	All sepsis	Tree-augmented naive Bayes	C, R, L
NOSEP-1 and NOSEP-NEW1 (Mahieu *et al*^112^ 2000, Mahieu *et al*^111^ 2002)	Belgium	LOS	Logistic regression	C, R, L
Okascharoen (Okascharoen *et al*^81^ 2005)	Thailand	LOS	Cox model	C, R, L
Perinatal Infection Risk Score (Takkar *et al*^116^ 1974)	India	All sepsis	ND	C, R
Philadelphia protocol (Baker *et al*^107^ 1993)	USA	Serious bacterial infection	Criteria-based without specific scoring	C, L
Pokhylko (Pokhylko *et al*^84^ 2020)	Ukraine	EOS	Logistic regression	C, R, L
PROM-Scoring (Afjeiee *et al*^26^ 2008)	Iran	All sepsis	Scoring system	C, R
Pukhtinskaya (Pukhtinskaya and Estrin^86^ 2021)	Russia	EOS	Decision tree	L
Rochester protocol (Powell *et al*^113^ 1990)	USA	Serious bacterial infection	Criteria-based without specific scoring	C, R, L
Rosenberg (Rosenberg *et al*^89^ 2010)	Bangladesh	LOS	Logistic regression	C
Selimovic (Selimovic *et al*^90^ 2010)	Bosnia and Herzegovina	EOS	Logistic regression	L
Septic screen (generic)	Various	All sepsis	NA	L
Shuai (Shuai *et al*^93^ 2022)	China	LOS	Logistic regression	R
Singh (Singh *et al*^95^ 2003)	India	LOS	Scoring system	C
STOPS tool (James *et al*^59^ 2021)	India	All sepsis	Scoring system	C, L
Weber (Weber *et al*^101^ 2003)	Ethiopia, the Gambia, Papua New Guinea and the Philippines	All sepsis, meningitis, pneumonia, or hypoxaemia	Logistic regression	C
Wu (Wu *et al*^102^ 2024)	China	All sepsis	Nomogram from logistic regression	R
Yadav (Yadav *et al*^103^ 2023)	India	All sepsis	Scoring system	L
Yin (Yin *et al*^104^ 2022)	China	Invasive bacterial infection	Decision tree from logistic regression	C, R, L
Zhang (Zhang *et al*^106^ 2023)	China	EOS	Logistic regression	C, R

Full table available in online supplemental appendix.

* Type of predictors included in final model.

C, clinical features; EOS, early-onset sepsis; HAI, healthcare-associated infection; L, laboratory tests; LOS, late-onset sepsis; ND, no data; R, risk factors.

In total, 24 252 neonates were included across all studies. The median number of participants per study was 151 (range 36 to 3303, IQR 200). Few studies restricted the study population based on gestational age or birth weight, with only four studies (5%) specifically investigating preterm neonates and five studies (6%) specifically investigating low or very low birth weight neonates. Most studies included neonates clinically suspected of sepsis or with specific maternal risk factors including chorioamnionitis (n*=*58, 71%).

Almost all studies included a positive blood and/or CSF culture in their outcome definition for sepsis (n*=*75, 91%). Of these, 18 (22% of all studies) also included clinical features or clinical suspicion of sepsis. One study used a consultant neonatologist’s clinical diagnosis of sepsis,^79^ one study used the International Classification of Diseases 10th Revision criteria for sepsis,^80^ and in three studies, the outcome was unclear.

### Model characteristics

The 82 included studies performed 109 evaluations validating 44 distinct models (table 3 and online supplemental table 2).^25^,^2835 36 43 49 50 52^ The most frequently validated model was the Hematological Scoring System by Rodwell *et al* (n*=*32, 39% of studies; including studies that made minor modifications to the original model).^115^ Most models were only validated in one study (n*=*34, 77% of models).

A total of 135 predictors of sepsis were included across all models, of which 82 were clinical parameters (signs, symptoms or risk factors) and 53 were laboratory parameters (online supplemental table 6). The median number of predictors per model was 6 (range 2 to 110, IQR 4). 14 models (32%) included only clinical parameters, 12 models (27%) included only laboratory parameters and 18 models (41%) included both. The most common laboratory parameters were white cell count (n*=*17 models, 39%), C-reactive protein (n*=*16 models, 36%) and platelet count (n*=*15 models, 34%). The most common clinical parameters were neonatal fever (n*=*13 models, 30%) and gestational age (n*=*11 models, 25%).

Most models were developed using logistic regression (n*=*16 models, 36%) (often with stepwise selection to select predictors) or consisted of a scoring system based on univariable predictor performance or literature review and expert opinion (n*=*10 models, 23%). Five models (11%) were developed using machine learning (ML) or artificial intelligence (AI)-based methods.^50 57 71 80 86^

### Model performance

Model performance was principally reported using sensitivity and/or specificity (with or without a confusion matrix); only four studies (5%) did not report either metric. 32 studies (39%) reported area under the receiver operating characteristic curve (AUC). Less frequent methods of quantifying performance included predictive values, likelihood ratios, accuracy, change in antibiotic use and mortality statistics. Across all 109 evaluations, median sensitivity was 81% (range 3% to 100%), and median specificity was 83% (range 11% to 100%).

#### Performance stratified by clinical vs laboratory parameters

In models containing both clinical and laboratory parameters (n*=*24 evaluations), median sensitivity was 73% (range 3% to 100%), and median specificity was 80% (range 18% to 98%). In models containing only clinical parameters (n*=*23 evaluations), median sensitivity was 67% (range 3% to 100%), and median specificity was 72% (range 11% to 99%). In models containing only laboratory parameters (n*=*62 evaluations), median sensitivity was 83% (range 9% to 100%), and median specificity was 86% (range 33% to 100%).

#### Performance stratified by target population

Models validated in a population with existing clinical suspicion of sepsis (due to signs and symptoms and/or presence of maternal risk factors for sepsis) had a median sensitivity of 82% (range 3% to 100%) and median specificity of 84% (range 18% to 100%) (n*=*75 evaluations). In comparison, models evaluated in the general neonatal population had a median sensitivity of 77% (range 15% to 100%) and median specificity of 80% (range 11% to 100%) (n*=*34 evaluations).

#### Performance stratified by sepsis timing

In models developed to diagnose both EOS and LOS (n*=*59 evaluations), median sensitivity was 82% (range 3% to 100%), and median specificity was 85% (range 33% to 100%). In models to diagnose only EOS, median sensitivity was 82% (range 9% to 100%), and median specificity was 82% (range 11% to 98%) (n*=*29 evaluations). In models to diagnose only LOS, median sensitivity was 57% (range 3% to 100%) and median specificity was 75% (range 18% to 99%) (n*=*21 evaluations).

## Discussion

Our scoping review provides a comprehensive overview of CPMs to diagnose neonatal sepsis in LMICs. Previous reviews of CPMs to diagnose neonatal sepsis have been published, but we included more studies than were identified in existing reviews despite our distinct focus on LMICs.^11^,^14^ The breadth of literature highlights the need for, and academic interest in, effective risk stratification for neonatal sepsis in LMICs. Several common themes emerged from our review, indicating gaps in the current literature and important lessons for future research. We make specific recommendations for future research in Box 2.

Box 2 **Recommendations for future research**

**Model development**

- Studies should focus on updating and validating existing clinical prediction models using data from low- and middle-income countries (LMICs) and low-resource settings. Better diagnostic tools could substantially reduce neonatal morbidity and mortality in these settings with the highest incidence of neonatal sepsis.
- Studies developing new models should use rigorous methodology and adhere to reporting standards to improve transparency and reproducibility. For multivariable clinical prediction models, authors should refer to the Transparent Reporting of a multivariable prediction model for Individual Prognosis Or Diagnosis+artificial intelligence (TRIPOD+AI) statement (www.tripod-statement.org).
- Studies should aim to develop and validate models that do not require laboratory parameters to improve applicability in many LMICs and low-resource settings.
- Studies should aim to develop and validate models to diagnose and stratify the risk of sepsis in populations of undifferentiated neonates at birth and/or at presentation to health facilities.

**Model validation**

- Clinicians and researchers should aim to develop a consensus definition of neonatal sepsis that can be applied across settings, including LMICs and low-resource settings. This would facilitate meaningful comparison of model performance between studies.
- Studies should consider reporting global metrics such as the area under the receiver operating characteristic curve or *C*-statistic to facilitate comparison of model performance between studies.
- Studies should clearly describe the population in which models have been validated and should aim to validate models across a range of gestational ages and birthweights.
- Studies should include qualitative and economic analyses to ensure clinical prediction models are acceptable, usable and affordable in the settings for which they were developed.
- As for model development, studies validating existing models should use rigorous methodology and adhere to the recommendations of the TRIPOD+AI statement to improve transparency and reproducibility.

First, 99% of studies were conducted in middle-income countries, with only one study including neonates born in a low-income country,^101^ and no studies conducted exclusively in a low-income country. Furthermore, fewer studies were conducted in the African region than any other WHO region, despite the high burden of neonatal sepsis and slower progress in neonatal mortality in these countries.^117 118^

Two-thirds of models required access to at least basic laboratory tests. Access to laboratory tests is limited in many low-resource settings or turnaround times are too long to usefully inform management.^119^ While use of laboratory parameters might offer additional accuracy—as suggested by the higher median sensitivity and specificity of these models compared to models with only clinical predictors—they may be less appropriate for some low-resource settings.

Most studies (78%) were conducted in neonatal intensive care or special care units, and only 29% were validated in a population of neonates that included infants without existing suspicion of sepsis. A substantial benefit of CPMs for neonatal sepsis in LMICs is their ability to promote early, targeted antibiotic therapy in an undifferentiated population of neonates, particularly in settings where care is led by health workers with less experience of neonatal medicine. This could reduce antibiotic overuse and the resultant antimicrobial resistance and adverse neonatal outcomes.^6^ While diagnostic decision support in high-risk neonates is useful, models are needed that can be applied at the time of birth to facilitate the rapid antimicrobial therapy required to reduce morbidity and mortality from EOS.^7^ Furthermore, few studies specifically evaluated predictive performance in preterm or low birth weight neonates. CPMs to diagnose neonatal sepsis should be evaluated across gestational ages and birthweights to ensure they perform well in the population of neonates most at risk of sepsis.

Some models were specifically developed to diagnose EOS or LOS, while many did not make this distinction. Restricting the outcome to EOS or LOS may allow more targeted risk stratification, but it is increasingly recognised that this conventional classification of neonatal sepsis may not be valid in LMICs given the high rates of healthcare-associated infections.^4^

Both reporting and results of model performance were highly variable between studies (eg, sensitivity ranged from 3% to 100% across all model evaluations). This variability likely arises not only from true differences in individual model performance, but also from heterogeneous reporting of model performance at arbitrary (or unreported) classification thresholds. Most studies only presented sensitivity and specificity; just over one-third of studies reported an easily comparable global metric such as AUC. Without consistent reporting of comparable metrics, it is impossible to disentangle these sources of variability. Therefore, the comparisons we present are illustrative of what is reported in the current literature, rather than suggestions of true differences in the performance of any specific type of model.

Few studies included practical discussion of how the proposed models could be integrated into routine neonatal care in LMICs. This includes how the probability or score can be calculated by healthcare workers (eg, using a paper proforma or a computer-based system). Most models were developed using logistic regression, but authors often simplify the final model to present a scoring system where each predictor is assigned an integer score. This approach is simpler for healthcare workers to use and interpret but can negatively impact the resulting predictions, particularly if continuous predictors are categorised.^8^ One solution could be to implement models using app or web-based interfaces for clinicians such as those proposed by Neotree,^120 121^ NoviGuide^122^ or NEST360^123^ to facilitate data entry and clinical decision support. This would also aid implementation of ML and AI-based prediction models. Potential benefits of ML and AI-based models include their ability to directly and automatically learn from data; greater flexibility for modelling non-linear associations and interactions; and ability to handle multimodal data compared with conventional regression.^124^ However, given that we only identified five CPMs using an ML/AI approach in our review, ML/AI-based models require rigorous validation in LMICs before they can be translated to clinical care.

Other clinical implications that are rarely discussed are how management decisions should be made based on model predictions, such as appropriate classification thresholds and how these can be incorporated into a wider sepsis management pathway within neonatal units. Arguably, the most well-known and validated model for EOS in high-resource settings is the Kaiser Permanente EOS calculator.^110^ This model has been recommended by some national guidelines (eg, the National Institute for Health and Care Excellence in the UK^7^) suggesting use of CPMs for neonatal sepsis is acceptable to clinicians in these settings. Implementation must be context-specific and will depend on the resources and clinical expertise available. For example, in settings with access to laboratory tests, approaches similar to those recommended by the Kaiser Permanente EOS calculator might be used,^110^ with specific classification thresholds triggering different management recommendations (eg, more frequent monitoring of vital signs, performing blood cultures or other tests, or starting empirical antibiotic therapy). In other settings, particularly where laboratory tests or specialist neonatal care are not available, CPMs may be used to make binary decisions on whether to start antibiotic therapy. Acceptable classification thresholds will depend on many contextual and subjective factors, including clinicians’ and families’ attitudes to risk, clinical workload and time pressures, and availability of resources including antibiotics, which may vary over time.^125^ Reporting of model performance, interpretation and clinical implications would be improved if authors refer to the TRIPOD statement, which specifically includes these items.^23^

Finally, three-quarters of models were only validated in one study in a LMIC, often only in the derivation cohort (apparent or internal validation). This may lead to overoptimistic performance due to overfitting.^8^ Several authors caution against the current focus on developing new models and advocate for further validation (including external validation) of promising existing models.^126^ Additionally, few studies included in our review assessed calibration performance of their models, which is an especially important consideration when models are intended for decision support.^127^

There are many barriers to developing and validating CPMs in low-resource settings. First, it is difficult to develop and validate diagnostic tools without access to a consistent, agreed reference standard. Despite the high incidence of neonatal sepsis globally, there is no internationally accepted consensus definition.^128^ Almost all studies included in our review (91%) used a positive blood and/or CSF culture in their outcome definition for neonatal sepsis. Even if researchers agree on this definition, there are many settings where microbiology facilities and consumables are inconsistently available or absent. Access to microbiological testing depends not only on the availability of accredited laboratories, but also on reliable access to consumables, including culture bottles and culture media, and timely temperature-controlled transport from the patient to laboratory. Expansion and reduced costs of newer molecular diagnostics may address this issue, but many techniques remain prohibitively expensive for facilities in LMICs.^129^ Where an outcome definition based on clinical diagnosis is used, this should be derived from agreed criteria, such as those proposed by the Brighton Collaborative,^130^ to improve inter-rater reliability and reduce between-study heterogeneity. Second, developing and validating CPMs requires access to complete, comprehensive and reliable clinical data. Many LMICs do not yet have sufficiently capable electronic health record systems from which these data can be extracted. Context-specific electronic health records that account for the practical and financial constraints of a low-resource setting may accelerate their adoption.^131^ Addressing these challenges would have wider benefits beyond the development and validation of CPMs. For example, improved access to microbiological testing in LMICs would enable individualised antibiotic therapy and better mapping of local epidemiology and antibiotic resistance to inform empirical antibiotic guidelines at the population level.

Several limitations of our review should be considered. First, we included only published studies. It may be that individual neonatal units or networks have developed their own diagnostic tools that are in local use but have not been published. Similarly, centres may use existing CPMs without publishing their validation data, particularly if these show poor model performance. Future systematic reviews of CPMs in LMICs could quantify publication bias through statistical assessment. Second, there was significant heterogeneity in study populations, definitions of sepsis and classification thresholds, which makes comparing model performance between studies particularly challenging. Given this heterogeneity, we were unable to conduct a meta-analysis of model performance even between studies assessing the same CPM. Therefore, we are unable to recommend specific models or types of models for clinicians caring for neonates in low-resource settings. A systematic review of model performance in specific populations and for specific definitions of sepsis would help to address this. Given the vast number of model variations presented in included studies, and the often arbitrarily selected classification thresholds at which model performance is reported, it is impractical for a single review to be completely comprehensive, and there is necessarily a degree of selective reporting in our review. Finally, we included only studies published in English or Spanish, though we are aware of the large number of LMICs that use an alternative official language. The nature of scientific publishing in LMICs means that studies are not always published in journals indexed in major biomedical databases. We identified 13 of the 82 included studies outwith our primary database searches through citation analysis and reference lists only (especially for studies published in India) and were unable to obtain full texts for 36 potentially eligible studies despite contacting authors. This raises the possibility of retrieval bias influencing our review.

## Conclusion

We conducted a comprehensive scoping review of CPMs to diagnose neonatal sepsis in LMICs. Despite the increasing number of studies published on this topic, we have highlighted several literature gaps, particularly a paucity of studies validating models in the lowest-income countries where neonatal sepsis is most prevalent, and models for the undifferentiated neonatal population that do not rely on laboratory tests. Furthermore, heterogeneity in study populations, definitions of sepsis and reporting of models inhibits meaningful comparison between studies and may hinder progress towards useful diagnostic tools.

## Supplementary material

10.1136/bmjgh-2024-017582 online supplemental file 1

10.1136/bmjgh-2024-017582 online supplemental file 2

10.1136/bmjgh-2024-017582 online supplemental file 3

10.1136/bmjgh-2024-017582 online supplemental file 4

10.1136/bmjgh-2024-017582 online supplemental material 1

## Data Availability

All data relevant to the study are included in the article or uploaded as supplementary information.
