# Supplementary material for: Clinical prediction models to diagnose neonatal sepsis in low-income and middle-income countries: a scoping review
Source: BMJ Glob Health. 2025 Apr 9;10(4):e017582. doi: 10.1136/bmjgh-2024-017582 (PMC12182008; doi:10.1136/bmjgh-2024-017582)
Supplement: online supplemental file 1 [file bmjgh-10-4-s001.pdf]

SUPPLEMENTARY APPENDIX

Appendix 1. Preferred Reporting Items for Systematic reviews and Meta-Analyses extension for Scoping Reviews (PRISMA-ScR) Checklist **2**

Appendix 2. Search strategies for each database **4**

Appendix 3. Data extraction form **7**

Appendix 4. Supplementary tables and figures **8**

Supplementary File. Full text decisions and exclusion reasons (.csv)

Supplementary File. Data extracted from included studies (.xlsx)

Supplementary File. Code used to analyse extracted data (.R)

Appendix 1. Preferred Reporting Items for Systematic reviews and Meta-Analyses extension for Scoping Reviews (PRISMA-ScR) Checklist

SECTION	ITEM	PRISMA-ScR CHECKLIST ITEM	REPORTED ON PAGE #
**TITLE**			
Title	1	Identify the report as a scoping review.	1
**ABSTRACT**			
Structured summary	2	Provide a structured summary that includes (as applicable): background, objectives, eligibility criteria, sources of evidence, charting methods, results, and conclusions that relate to the review questions and objectives.	1
**INTRODUCTION**			
Rationale	3	Describe the rationale for the review in the context of what is already known. Explain why the review questions/objectives lend themselves to a scoping review approach.	3
Objectives	4	Provide an explicit statement of the questions and objectives being addressed with reference to their key elements (e.g., population or participants, concepts, and context) or other relevant key elements used to conceptualize the review questions and/or objectives.	3
**METHODS**			
Protocol and registration	5	Indicate whether a review protocol exists; state if and where it can be accessed (e.g., a Web address); and if available, provide registration information, including the registration number.	5
Eligibility criteria	6	Specify characteristics of the sources of evidence used as eligibility criteria (e.g., years considered, language, and publication status), and provide a rationale.	Table 1
Information sources	7	Describe all information sources in the search (e.g., databases with dates of coverage and contact with authors to identify additional sources), as well as the date the most recent search was executed.	5
Search	8	Present the full electronic search strategy for at least 1 database, including any limits used, such that it could be repeated.	Appendix 2
Selection of sources of evidence	9	State the process for selecting sources of evidence (i.e., screening and eligibility) included in the scoping review.	5
Data charting process	10	Describe the methods of charting data from the included sources of evidence (e.g., calibrated forms or forms that have been tested by the team before their use, and whether data charting was done independently or in duplicate) and any processes for obtaining and confirming data from investigators.	6

Data items	11	List and define all variables for which data were sought and any assumptions and simplifications made.	Appendix 3
Critical appraisal of individual sources of evidence	12	If done, provide a rationale for conducting a critical appraisal of included sources of evidence; describe the methods used and how this information was used in any data synthesis (if appropriate).	Not applicable
Synthesis of results	13	Describe the methods of handling and summarizing the data that were charted.	6
**RESULTS**			
Selection of sources of evidence	14	Give numbers of sources of evidence screened, assessed for eligibility, and included in the review, with reasons for exclusions at each stage, ideally using a flow diagram.	Figure 1
Characteristics of sources of evidence	15	For each source of evidence, present characteristics for which data were charted and provide the citations.	6-7
Critical appraisal within sources of evidence	16	If done, present data on critical appraisal of included sources of evidence (see item 12).	Not applicable
Results of individual sources of evidence	17	For each included source of evidence, present the relevant data that were charted that relate to the review questions and objectives.	Table 2 and 3
Synthesis of results	18	Summarize and/or present the charting results as they relate to the review questions and objectives.	7-8
**DISCUSSION**			
Summary of evidence	19	Summarize the main results (including an overview of concepts, themes, and types of evidence available), link to the review questions and objectives, and consider the relevance to key groups.	8-10
Limitations	20	Discuss the limitations of the scoping review process.	10
Conclusions	21	Provide a general interpretation of the results with respect to the review questions and objectives, as well as potential implications and/or next steps.	11
**FUNDING**			
Funding	22	Describe sources of funding for the included sources of evidence, as well as sources of funding for the scoping review. Describe the role of the funders of the scoping review.	12

Adapted from: Tricco AC, Lillie E, Zarin W, O'Brien KK, Colquhoun H, Levac D, et al. PRISMA Extension for Scoping Reviews (PRISMAScR): Checklist and Explanation. Ann Intern Med. 2018;169:467–473. doi: 10.7326/M18-0850.

Appendix 2. Search strategies for each database

Ovid MEDLINE

Ovid MEDLINE(R) and Epub Ahead of Print, In-Process & Other Non-Indexed Citations and Daily

1	exp Infant, Newborn/
2	(neonat* or newborn* or new born* or baby or babies or premature or preterm or infant* or low birth weight or LBW or VLBW or ELBW or NICU*).ti,ab,kw.
3	1 or 2
4	exp Sepsis/
5	(sepsis or septic* or bacter?emia).ti,ab,kw.
6	4 or 5
7	Decision Support Techniques/ or Neonatal Screening/
8	((predict* or diagnos* or screen* or identif* or manag*) adj5 (model* or rule* or scor* or tool* or algorithm* or decision tree* or pathway* or calculator*)).ti,ab,kw.
9	7 or 8
10	3 and 6 and 9

Ovid Embase

1	Newborn/
2	(neonat* or newborn* or new born* or baby or babies or premature or preterm or infant* or low birth weight or LBW or VLBW or ELBW or NICU*).ti,ab,kw.
3	1 or 2
4	exp sepsis/
5	(sepsis or septic* or bacter?emia).ti,ab,kw.
6	4 or 5
7	exp decision support system/ or newborn screening/
8	((predict* or diagnos* or screen* or identif* or manag*) adj5 (model* or rule* or scor* or tool* or algorithm* or decision tree* or pathway* or calculator*)).ti,ab,kw.
9	7 or 8
10	3 and 6 and 9

Scopus

TITLE-ABS-KEY((neonat* OR newborn* OR "new born*" OR baby OR babies OR premature OR preterm OR infant* OR "low birth weight" OR lbw OR vlbw OR elbw OR nicu*) AND (sepsis OR septic* OR bacter?emia) AND ((predict* OR diagnos* OR screen* OR identif* or manag*) W/5 (model* OR rule* OR scor* OR tool* OR algorithm* OR "decision tree*" OR pathway* OR calculator*)))

Web of Science
Core Collection

TS=((neonat* OR newborn* OR "new born*" OR baby OR babies OR premature OR preterm OR infant* OR "low birth weight" OR lbw OR vlbw OR elbw OR nicu*) AND (sepsis OR septic* OR bacter$emia) AND ((predict* OR diagnos* OR screen* OR identif* or manag*) NEAR/5 (model* OR rule* OR scor* OR tool* OR algorithm* OR "decision tree*" OR pathway* OR calculator*)))

Global Index Medicus
All indexes: LILCAS (Americas), WPRIM (Western Pacific), IMSEAR (South-East Asia), IMEMR (Eastern Mediterranean), AIM (Africa)

((mh:("Infant, Newborn")) OR (tw:(neonat* OR newborn* OR "new born*" OR baby OR babies OR premature OR preterm OR infant* OR "low birth weight" OR lbw OR vlbw OR elbw OR nicu*))) AND ((mh:(“Sepsis”)) OR (tw:(sepsis OR septic* OR bacter*emia))) AND ((mh:(“Decision Support Systems, Clinical” OR “Neonatal Screening”)) OR (tw:(model* OR rule* OR scor* OR tool* OR algorithm* OR "decision tree*" OR pathway* OR calculator*)))

Cochrane Library
Cochrane Database of Systematic reviews and Cochrane Central Register of Controlled Trials

1	MeSH descriptor: [Infant, Newborn] explode all trees
2	(neonat* or newborn* or “new born*” or baby or babies or premature or preterm or infant* or “low birth weight” or LBW or VLBW or ELBW or NICU*):ti,ab,kw
3	#1or#2
4	MeSH descriptor: [Sepsis] explode all trees
5	(sepsis or septic* or bacter*emia):ti,ab,kw
6	#4or#5
7	MeSH descriptor: [Decision Support Systems, Clinical] explode all trees
8	MeSH descriptor: [Neonatal Screening] explode all trees
9	((predict* or diagnos* or screen* or identif* or manag* or estimat*) NEAR/5 (model* or rule* or scor* or tool* or algorithm* or “decision tree*” or pathway* or calculator*)):ti,ab,kw
10	#7 or #8 or #9
11	#3 and #6 and #10

Note on search strategy development

We were interested in capturing studies that validate models to diagnose neonatal sepsis regardless of how this was defined by individual study authors. When developing the search strategy, we reviewed the MeSH terms and keywords used to index known relevant studies (including reviews) on prediction models for neonatal sepsis in Ovid MEDLINE. Therefore, we only included the MeSH term 'Sepsis' and the keywords 'sepsis' or 'bacteraemia' to capture our outcome concept. Notably, we did not identify 'meningitis' as an important keyword in known relevant studies as no study explored sepsis and meningitis separately. Hence, we chose not to include 'meningitis' in our search strategy even though many definitions of neonatal sepsis include a positive cerebrospinal fluid culture.

Appendix 3. Data extraction form

Study characteristics	
Study ID	Title, author, year
Country	
Context	E.g. NICU/SCBU admissions, all babies born at study site, presenting to emergency care services
Number of included participants	
Characteristics of included participants	E.g. preterm, low birthweight
Objectives	
Outcome definition	E.g. blood culture, CSF culture, clinical diagnosis
Study results	
Name of model	
Modelling methods	E.g. logistic regression, scoring system
Predictors in final model	Signs, symptoms, risk factors, laboratory parameters
Model performance	Sensitivity, specificity, predictive values, likelihood ratios, AUC, accuracy, acceptability, antibiotic use, mortality, any other factors

Appendix 4. Supplementary tables and figures

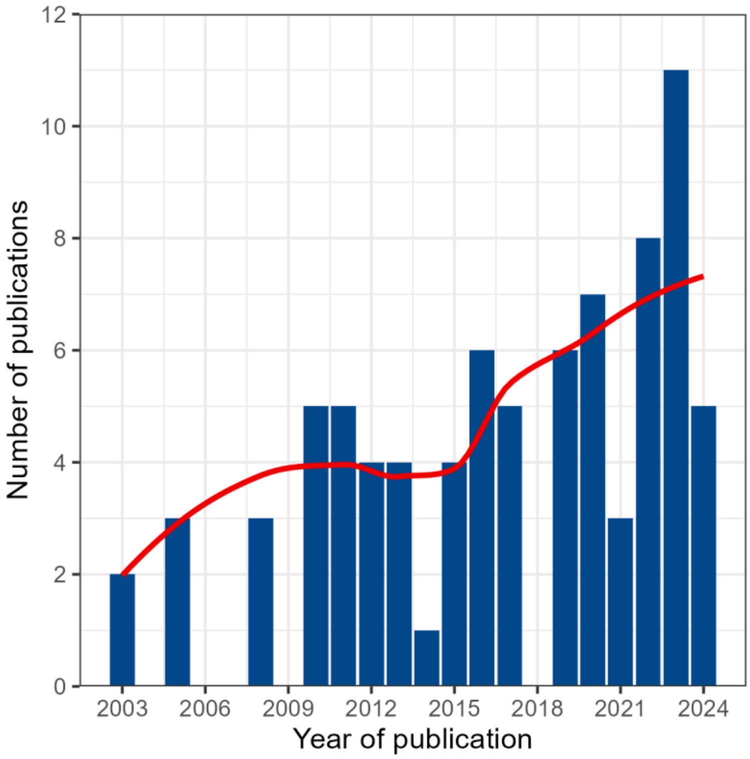

Supplemental Figure 1. Number of studies included in our review by year of publication. Red line represents a local regression (loess) model fitted on the yearly publication counts.

Supplemental Table 1. Summary of included studies and model performance.

Model	Study, country	Population	No. participants (with outcome)	Outcome definition	Sensitivity	Specificity	AUC	Additional metrics and comments
Abiramalatha	Abiramalatha 2016, India	All births, at risk or suspected of sepsis	600 (240)	Blood culture	90.8%	99.7%	ND	Threshold unclear
Ahire	Ahire 2022, India	NICU, high sepsis risk	80 (57)	Blood culture	94.5%	80.0%	ND	Threshold unclear
Ahmed	Ahmed 2005, Pakistan	NICU, suspected sepsis	200 (28)	Blood culture	96.4-100%	72.0-92.0%	ND	Various 3 test combinations
American Academy of Pediatrics (AAP)	Beandda 2019, the Philippines	All births, ≥34 weeks, treated for sepsis	330 (11)	Blood culture	54.6%	72.2%	ND	Threshold unclear
Bekhof	Lloyd 2022, South Africa	NICU, very low birthweight	841 (224) episodes	Blood culture	55%	71%	ND	Threshold ≥1 sign positive
Boston protocol	Bulbul 2020, Turkey	NICU, ≥35 weeks, fever ≥38°C	328 (126)	Blood, CSF, sputum or urine culture	61.6%	81.7%	ND	Threshold ≥1 criterion positive
Celik	Celik 2013, Turkey	NICU	304 (206)	Clinical diagnosis ± blood culture	95.9%	91.2%	AUC 0.98 (95% CI 0.95-0.99)	Threshold 0.2429
Fitriana	Fitriana 2023, Indonesia	NICU, all admitted	162 (15)	Blood culture	80%	47%	AUC 0.762	Threshold ≥2.5
He	He 2017, China	NICU, suspected sepsis	151 (68)	Blood or CSF culture	42.7%	94.0%	AUC 0.83 (95% CI 0.77-0.89)	Threshold 3 criteria positive
Helguera-Repetto	Helguera-Repetto 2020, Mexico	NICU	238 (106)	Blood culture	93.3%	80.0%	AUC 0.94 (95% CI 0.86-1.00)	Threshold unclear
Hematological Scoring System (HSS)	Amir 2023, India	NICU, suspected sepsis	70 (9)	Blood culture	100.0%	75.4%	ND	Threshold ≥4
	Annam 2015, India	All births, term	153 (59)	Blood culture	100%	78.7%	ND	Threshold ≥4
	Bhalodia 2017, India	NICU, suspected sepsis	150 (48)	Blood culture	100%	100%	ND	Threshold ≥5
	Debroy 2016, India	NICU, suspected sepsis	40 (10)	Blood culture	80%	90%	ND	Threshold ≥5
	Derbala 2017, Egypt	NICU, suspected sepsis	200 (80)	ND	95.0%	96.7%	AUC 0.95 (95% CI 0.94-1.00)	Threshold ≥4
	Dutta 2016, India	NICU, suspected sepsis	210 (48)	Blood culture	100%	69.8%	ND	Threshold ≥5
	El-Said 2024, Egypt	NICU, culture proven sepsis and negative controls	225 (107)	Blood culture	87.85%	94.92%	AUC 0.964	Threshold ≥3
	Gupta 2011, India	NICU, at risk or suspected of sepsis	80 (44)	Clinical diagnosis ± blood culture	ND	ND	ND	75% with clinical sepsis scored ≥5

	Ibrahim 2023, Egypt	NICU, suspected sepsis and non-septic controls	81 (51)	Clinical diagnosis ± blood culture	72.6%	100.0%	AUC 0.863 (95% CI 0.768-0.929)	Threshold >1
	Khair 2012, India	NICU, suspected sepsis	100 (12)	Blood culture	75%	87%	ND	Threshold ≥5
	Krishnamurthy 2017, India	NICU, full blood count performed at admission	75 (25)	Clinical diagnosis ± blood culture	80%	70%	ND	Threshold ≥3
	Liestiadi 2017, Indonesia	NICU, suspected sepsis	43 (14)	Blood culture	100.0%	82.7%	AUC 0.946	Threshold ≥4
	Makkar 2013, India	Presenting to hospital, suspected sepsis	110 (42)	Clinical diagnosis ± blood culture	Preterm: 92.3%<br>Term: 75.0%	Preterm: 86.4%<br>Term: 77.8%	ND	Threshold unclear
	Malini 2016, India	NICU, suspected sepsis	200 (116)	Blood culture	95%	86%	ND	Threshold ≥5
	Manvitha 2023, India	NICU, suspected sepsis	300 (185) samples	Blood culture and CRP	96.55%	59.23%	ND	Threshold ≥3
	Meirina 2015, Indonesia	NICU, suspected sepsis	40 (10)	Blood culture	80%	90%	AUC 0.90 (95% CI 0.80-1.00)	Threshold ≥4
	Mishra 2019, India	NICU, suspected sepsis	50 (18)	Blood culture	88.9%	100.0%	ND	Threshold ≥5
	Nair 2020, India	NICU, suspected sepsis	80 (12)	Blood culture	41%	92%	ND	Threshold unclear
	Narasimha 2011, India	NICU, at risk or suspected of sepsis	50 (12)	Blood culture	100%	66%	ND	Threshold ≥5
	Padhy 2023, India	NICU, suspected sepsis and non-septic controls	350 (146)	Clinical diagnosis and SIRS	79%	39%	ND	Threshold ≥3
	Pramana 2016, Indonesia	All births, at risk or suspected of sepsis	62 (21)	Blood culture	80.9%	92.7%	ND	Threshold ≥5
	Shah 2019, India	NICU, suspected sepsis	100 (19)	Blood culture	10.5%	100%	ND	Threshold ≥5
	Shukla 2023, India	NICU, at risk of sepsis	100 (35)	Clinical diagnosis ± blood culture	63%	88%	ND	Threshold ≥3
	de Souza 2015, Brazil	All births, suspected sepsis	50 (16)	Blood culture	15%	97%	AUC 0.61	Threshold >3
HSS (cord blood)	Himasree 2024, India	All births, at risk of sepsis	100 (56)	Blood culture	100.0%	54.7%	ND	Threshold ≥2
	Shukla 2023, India	NICU, at risk of sepsis	100 (35)	Clinical diagnosis ± blood culture	74%	92%	ND	Threshold ≥3
HSS<br>(+) CRP	Amir 2023, India	NICU, suspected sepsis	70 (9)	Blood culture	100.0%	86.9%	ND	Threshold ≥4 plus CRP
	Khair 2012, India	NICU, suspected sepsis	100 (12)	Blood culture	75%	85%	ND	Threshold ≥4 plus CRP
	Nabi 2019, Bangladesh	All births	140 (22)	Blood culture	93%	55-57%	ND	Various 3 test combinations

HSS<br>(+) PCR	Godoy Torales 2020, Paraguay	NICU, term, chorioamnionitis	71 (11)	Clinical diagnosis ± blood culture	At mean 3.8 hours of life: 9.0%;<br>At median 45.5 hours of life: 30.0%	At mean 3.8 hours of life: 98.3%;<br>At median 45.5 hours of life: 96.1%	ND	Threshold ≥3
HSS<br>(+) CRP, micro-ESR	Amir 2023, India	NICU, suspected sepsis	70 (9)	Blood culture	100.0%	91.8%	ND	Threshold ≥4 plus CRP and micro-ESR
HSS<br>(+) nucleated RBCs<br>(-) I:M ratio<br>('Sepscore')	Padhy 2023, India	NICU, suspected sepsis and non-septic controls	350 (146)	Clinical diagnosis and SIRS	68%	61%	ND	Threshold ≥3
	Sharma 2024, India	Unclear, at risk or suspected of sepsis	108 (35)	Blood culture	82.85%	91.78%	AUC 0.6083	Threshold ≥3
HSS<br>(+) nucleated RBCs<br>(-) I:M ratio, immature PMN count<br>('modified HSS')	Ibrahim 2023, Egypt	NICU, suspected sepsis and non-septic controls	81 (51)	Clinical diagnosis ± blood culture	76.5%	100.0%	AUC 0.882 (95% CI 0.792 to 0.943)	Threshold >1
	Krishnamurthy 2017, India	NICU, full blood count performed at admission	75 (25)	Clinical diagnosis ± blood culture	84%	82%	ND	Threshold ≥3
HSS<br>(+) CRP, CD64<br>(-) PMN changes	Mohamed 2012, Egypt	NICU, suspected sepsis and healthy term controls	36 (13)	Clinical diagnosis and blood, CSF or urine culture	100.0%	82.6%	ND	Threshold ≥3
HSS<br>(+) cord CRP, 48-hour CRP<br>(-) I:T ratio	Varughese 2019, India	NICU, ≥34 weeks	550 (17)	Blood culture	94.1%	89.9%	ND	Threshold ≥5
HSS<br>(+) nucleated RBCs<br>(-) I:M ratio, PMN changes	Chitra 2022, Indonesia	NICU, suspected sepsis	95 (28)	Blood culture	82.1%	83.6%	AUC 0.849	Threshold ≥3
HSS<br>(+) nucleated RBCs, MPV, PDW<br>(-) I:M ratio, PMN changes	Chitra 2022, Indonesia	NICU, suspected sepsis	95 (28)	Blood culture	92.9%	83.6%	AUC 0.917	Threshold ≥3

HSS (+) CRP, partial blood culture result ('ANVISA handbook protocol')	Pinto 2013, Brazil	NICU, very low birthweight	136 (129)	Clinical diagnosis and blood culture	ND	ND	ND	Significant decrease in antibiotic regimens, days of antibiotics, overall mortality and infection-related mortality
Hu	Hu 2021, China	NICU, preterm	156 (65)	Blood or CSF culture	90.8%	97.8%	AUC 0.97 (95% CI 0.94-1.00)	Threshold unclear
Huang	Huang 2020, China	NICU, preterm	1708 (130)	Clinical diagnosis and blood culture	45.3%	92.3%	AUC 0.83 (95% CI 0.78-0.89)	Threshold unclear
	Shuai 2022, China	NICU, preterm, admitted within 24 hours of birth for >7 days	119 (ND)	Clinical diagnosis and blood culture	44.1%	71.7%	AUC 0.61 (95% CI 0.51-0.72)	Threshold 0.164
Husada EOS	Husada 2010, Thailand	ND	180 (45)	ND	73.3%	84.4%	AUC 0.88	Threshold ≥1
Husada LOS	Husada 2010, Thailand	NICU	208 (52)	Blood culture	88.5%	90.4%	AUC 0.96	Threshold ≥2
Iqbal	Iqbal 2024, India	NICU, clinically diagnosed with sepsis	388 (184)	Blood culture	ND	ND	AUC 0.994	Threshold 0.07396
Istanbul protocol	Bulbul 2020, Turkey	NICU, ≥35 weeks, fever ≥38°C	328 (126)	Blood, CSF, sputum or urine culture	81.7%	65.0%	ND	Threshold ≥1 criterion positive
Kaiser Permanente EOS Calculator	Al-Lawama 2019, Jordan	NICU, PROM ≥18 hours, ≥34 weeks	176 (32)	Clinical diagnosis ± blood culture	ND	ND	ND	Newborns with sepsis fit into "ill appearing" category with significantly higher proportion
	Beandda 2019, the Philippines	All births, ≥34 weeks, treated for sepsis	330 (11)	Blood culture	54.6%	87.8%	ND	
	He 2020, China	NICU, ≥34 weeks	501 (353)	Blood culture	81.2%	93.9%	AUC 0.91 (95% CI 0.88-0.94)	
	Ikhsaniatun 2022, Indonesia	NICU, ≥34 weeks, clinical diagnosis of sepsis using WHO criteria	82 (21)	Blood culture	85.7%	44.3%	ND	Positive blood culture RR 3.46 (95% CI 1.11-10.78); antibiotic use decreased by 36.6%

Kar	Kar 2010, India	NICU, suspected sepsis at ≥3 days of life	210 (94) events	Clinical diagnosis and blood or CSF culture	3%	99%	ND	Threshold ≥4
Matsushita	Matsushita 2022, Brazil	NICU	1181 (175) samples	Blood culture	8-59%	ND	AUC 0.695-0.775	Various machine learning models
Mondal	Mondal 2012, India	NICU, suspected sepsis	62 (38)	Blood culture	84%	84%	ND	Threshold ≥2 tests positive
Neal	Neal 2023, Zimbabwe	NICU, ≥32 weeks	2628 (297)	Clinical diagnosis	95%	11%	AUC 0.74 (95% CI 0.70-0.77)	Threshold 0.034
NeoHoP	Lloyd 2023, South Africa	NICU, very low birthweight, admitted >72 hours with suspected HAI	552 (215) episodes	Clinical diagnosis ± blood culture	54.2%	96.4%	AUC 0.868 (95% CI 0.837-0.900)	Threshold ≥2
Nguyen	Nguyen 2023, China	PICU, all admitted <30 days	ND (ND)	ICD-10 diagnostic criteria	33.8%	96.1%	AUC 0.783 (95% CI 0.716–0.850)	Threshold unclear
NOSEP-1	Lloyd 2022, South Africa	NICU, very low birthweight	841 (224)	Blood culture	65%	75%	ND	Threshold ≥8
	Reyna-Figueroa 2005, Mexico	NICU, preterm, very low birthweight, suspected sepsis	101 (51)	Blood culture and ≥2 SIRS features	62.7%	70.0%	AUC 0.68 (95% CI 0.57-0.78)	Threshold ≥8
NOSEP-NEW1	Lloyd 2022, South Africa	NICU, very low birthweight	841 (224)	Blood culture	17%	97%	ND	Threshold ≥11 NOSEP-1 but with different cutoffs for variables
Okascharoen	Okascharoen 2005, Thailand	All births, hospitalised >72 hours	73 (25)	Clinical diagnosis and positive blood, CSF, pleural, bone, joint or urine culture	92%	56%	AUC 0.80 (95% CI 0.69-0.90)	Threshold ≥4
	Raguindin 2014, the Philippines	NICU, suspected LOS	119 (60)	Blood culture	83.3%	61.0%	AUC 0.75 (95% CI 0.66-0.84)	Threshold ≥6
Perinatal Infection Risk Score	Hassan 2016, India	All births, at risk of sepsis	100 (63)	Blood culture	20.6%	92.0%	ND	Threshold ≥6
	Sriram 2011, India	NICU, suspected sepsis	115 (58)	Blood culture	43.1%	57.9%	ND	Threshold ≥6
Philadelphia protocol	Bulbul 2020, Turkey	NICU, ≥35 weeks, fever ≥38°C	328 (126)	Blood, CSF, sputum or urine culture	67.7%	79.9%	ND	Threshold ≥1 criterion positive
Pokhylko	Pokhylko 2020, Ukraine	NICU, low birthweight	152 (121)	Blood culture	82.20%	93.55%	AUC 0.94	Threshold unclear

PROM-Scoring	Afjeiee 2008, Iran	All births, PROM ≥18 hours	270 (79)	Blood culture	100%	ND	ND	Threshold unclear
Pukhtinskaya	Pukhtinskaya 2021, Russia	NICU, term, admitted for mechanical ventilation within 48 hours of birth	200 (45)	Unclear	97.06%	94.67%	ND	Threshold unclear
Rochester protocol	Bulbul 2020, Turkey	NICU, ≥35 weeks, fever ≥38°C	328 (126)	Blood, CSF, sputum or urine culture	47.6%	72.0%	ND	Threshold ≥1 criterion positive
	Zarkesh 2011, Iran	Presenting to emergency department and admitted to NICU, term, febrile	202 (38)	Clinical diagnosis and blood, CSF, urine or stool culture	2.63%	62.2%	ND	Threshold ≥1 criterion positive
Rosenberg	Lloyd 2022, South Africa	NICU, very low birthweight	841 (224)	Blood culture	17%	95%	ND	Threshold ≥2
	Rosenberg 2010, Bangladesh	NICU, ≤33 weeks	193 (105)	Clinical diagnosis ± blood culture	15.2%	96.6%	ND	Threshold ≥3
Selimovic	Selimovic 2010, Bosnia & Herzegovina	All births, suspected sepsis	341 (199)	Clinical diagnosis ± blood culture	73%	89%	AUC 0.87	Threshold 0.503
Septic screen	Buch 2011, India	NICU, suspected sepsis	120 (65)	Blood culture	81.5%	94.6%	ND	ANC, I:T ratio, platelet count, CRP, micro-ESR
	Gupta 2022, India	NICU, ≥34 weeks, suspected sepsis	300 (38)	Blood culture	92.1%	34.0%	ND	CRP, micro-ESR, WCC, ANC, I:T ratio
	Hassan 2016, India	All births, at risk of sepsis	100 (63)	Blood culture	81.0%	94.6%	ND	Threshold ≥2 tests from WCC, ANC, I:T ratio, platelet count, CRP, micro-ESR
	Jadhav 2013, India	NICU, suspected sepsis	115 (75)	Blood culture	100.0%	62.5%	ND	Threshold ≥2 tests from WCC, ANC, I:T ratio, CRP
	Kudawla 2008, India	NICU, low birthweight, suspected sepsis	220 (60) events	Clinical diagnosis and blood culture	48%	70%	ND	Threshold ≥2 tests from WCC, CRP, micro-ESR, I:T ratio

	Mahale 2010, India	NICU, suspected sepsis	125 (28)	Blood culture	Mild/moderate : 38%<br>Severe: 25%	Mild/moderate : 81%<br>Severe: 77%	ND	Threshold ≥2 tests from CRP, ANC, I:T ratio, micro-ESR; illness severity SNAPPE-II
	Sriram 2011, India	NICU, suspected sepsis	115 (58)	Blood culture	65.4%	81.1%	ND	Threshold ≥3 tests from CRP, WCC, ANC, I:T ratio, platelet count, micro-ESR, buffy coat smear
	Swarnkar 2012, India	NICU	189 (37)	Blood culture	66.7%	79.0%	ND	Threshold ≥3 tests from CRP, WCC, ANC, I:T ratio, micro-ESR, buffy coat smear
	Thermiany 2008, Indonesia	NICU, suspected sepsis	126 (56)	Blood culture	85.7%	97.1%	ND	Threshold ≥2 tests from WCC, platelet count, I:T ratio
	Vinay 2015, India	NICU, suspected sepsis	60 (48)	Blood culture	77%	41%	ND	Threshold ≥2 tests from WCC, ANC, CRP, I:T ratio, platelet count, micro-ESR
	Yadav 2023, India	NICU, suspected sepsis	300 (174)	Blood culture or ≥2 positive inflammatory markers	58%	32.62%	ND	Threshold ≥2 tests from WCC, I:T ratio, ANC, CRP
Shuai	Shuai 2022, China	NICU, preterm, admitted within 24 hours of birth for >7 days	119 (ND)	Clinical diagnosis and blood culture	67.8%	75.0%	AUC 0.80 (95% CI 0.72-0.88)	Threshold 0.539
Singh	Singh 2003, India	NICU	105 (30) events	Blood or CSF culture	87%	29%	ND	Threshold ≥1
	Kudawla 2008, India	NICU, low birthweight, suspected sepsis	220 (60) events	Clinical diagnosis and blood culture	90.0%	22.5%	ND	Threshold ≥1
	Rosenberg 2010, Bangladesh	NICU, ≤33 weeks	193 (105)	Clinical diagnosis ± blood culture	56.6%	52.1%	ND	Threshold ≥3
	Lloyd 2022, South Africa	NICU, very low birthweight	841 (224)	Blood culture	32%	76%	ND	Threshold ≥3

Singh<br>(+) septic screen	Kudawla 2008, India	NICU, low birthweight, suspected sepsis	220 (60) events	Clinical diagnosis and blood culture	95.0%	18.1%	ND	Threshold ≥1 and/or septic screen positive
STOPS tool	James 2021, India	NICU, at risk or suspected of sepsis	EOS: 330 (86)<br>LOS: 50 (20)	Clinical diagnosis and blood culture	EOS: 90.5%<br>LOS: 100.0%	EOS: 28.5%<br>LOS: 90.0%	ND	Strategy 3 for EOS,<br>Strategy 2 for LOS; 33% decrease in antibiotic use for EOS, 54% for LOS
Weber	Weber 2003, Ethiopia, the Gambia, Papua New Guinea & the Philippines	Presenting to study sites, <60 days of age	3303 (120)	Blood culture	0-6 days: 87%;<br>7-59 days: 66%	0-6 days: 41%;<br>7-59 days: 79%	ND	Threshold any 2 signs positive
Wu	Wu 2024, China	All births, term, vaginal delivery, mothers colonised with GBS	339 (84)	Clinical diagnosis	68.6%	61.9%	AUC 0.711 (95% CI 0.592-0.808)	Threshold 0.305
Yadav	Yadav 2023, India	NICU, suspected sepsis	300 (174)	Blood culture or ≥2 positive inflammatory markers	41.36%	60.43%	ND	Threshold ≥2 tests positive
Yin	Yin 2022, China	NICU, term, suspected sepsis	1053 (166)	Blood culture and metagenomic next-generation sequencing of blood or CSF	95.3%	ND	ND	Accuracy 98.7%
Zhang	Zhang 2023, China	NICU, suspected sepsis	111 (ND)	Blood culture	98.6%	95.0%	AUC 0.721 (95% CI 0.587-0.854)	Threshold 0.856

‘Suspected sepsis’ refers to a population of neonates investigated for sepsis due to suggestive clinical features or risk factors. ‘Clinical diagnosis’ refers to a documented outcome of sepsis by a health worker or study investigator, which may be based on clinical features, risk factors, and/or non-microbiological laboratory parameters. ‘Threshold’ refers to the classification threshold at which model performance was presented (i.e. the threshold for determining a positive prediction of sepsis). ANC = absolute neutrophil count; ANVISA = Agência Nacional de Vigilância Sanitária; AUC = area under the curve; CD = cluster of differentiation; CI = confidence interval; CRP = C-reactive protein; CSF = cerebrospinal fluid; EOS = early-onset sepsis; ESR = erythrocyte sedimentation rate; HAI = healthcare-associated infection; ICD-10 = International Classification of Diseases 10th revision; I:M ratio = immature to mature neutrophil ratio; I:T ratio = immature to total neutrophil ratio; LOS = late-onset sepsis; MPV = mean platelet volume; ND = no data; NICU = neonatal intensive care unit; PCR = polymerase chain reaction; PDW = platelet distribution width; PICU = paediatric intensive care unit; PMN = polymorphonuclear neutrophil; PROM = premature rupture of membranes; RBC = red blood cell; RR = relative risk; SIRS = systemic inflammatory response syndrome; WCC = white cell count; WHO = World Health Organization.

Supplemental Table 2. Summary of model characteristics.

Model (derivation study)	Country of derivation cohort	Outcome	Modelling methods	Predictors in final model		
				**Clinical features**	**Risk factors**	**Laboratory tests**
Abiramalatha (Abiramalatha 2016)	India	All sepsis	Scoring system, ROC curves to determine cutoff values			WCC, platelet count, lower median angle light scatter, mean neutrophil volume
Ahire (Ahire 2022)	India	All sepsis	Scoring system			CRP, WCC, platelet count, neutrophil count
Ahmed (Ahmed 2005)	Pakistan	All sepsis	Scoring system, literature and clinical knowledge			CRP, neutrophil count, platelet count, gastric aspirate cytology, cytoplasmic vacuolation (3 test combinations)
American Academy of Pediatrics (AAP) (Puopolo 2018)	USA	EOS	Criteria-based without specific scoring	Ill appearance (multifactorial)	Chorioamnionitis	
Bekhof (Bekhof 2013)	The Netherlands	LOS	Nomogram from logistic regression, backward elimination	Increased respiratory support, capillary refill time, grey skin	Central venous catheter	
Boston protocol (Baskin 1992)	USA	Serious bacterial infection	Criteria-based without specific scoring	Fever ≥38°C, appearance, dehydration, focal signs of infection	Recent immunisation, recent antibiotics	WCC, CSF analysis, urinalysis, CXR
Celik (Celik 2013)	Turkey	All sepsis	Markov state models, modified stepwise selection			Mean neutrophil volume, volume distribution width, interleukin-6, CRP
Fitriana (Fitriana 2023)	Indonesia	EOS	Logistic regression		Prematurity, PROM, foul liquor, sex	
Hematological Scoring System (Rodwell 1988)	Australia	All sepsis	Scoring system, univariable predictor performance			WCC, PMN count, immature PMN count, I:T ratio, I:M ratio, platelet count, PMN degenerative changes
He (He 2017)	China	EOS	Logistic regression, stepwise selection			Interleukin-27, procalcitonin, CRP

Helguera-Repetto (Helguera-Repetto 2020)	Mexico	All sepsis	Neural network	Fever >37.5°C, hypothermia <35.5°C, tachycardia, tachypnoea, bradycardia, bradypnoea, apnoeas	PROM, chorioamnionitis, maternal age, maternal morbidity, cervicovaginitis, UTI, sex, gestational age, birthweight, foetal morbidity, catheter, mechanical ventilation	WCC, platelet count, neutrophil count, band cells, % bands, I:T ratio
Hu (Hu 2021)	China	EOS	Logistic regression, backward elimination	Apgar score		CRP, procalcitonin, interleukin-6
Huang (Huang 2020)	China	LOS	Nomogram from logistic regression, backward elimination		Birthweight, intubation, umbilical venous catheter duration	Thyroid function
Husada EOS (Husada 2010)	Thailand	EOS	Logistic regression	Oxygen requirement, poor feeding	Length of admission pre-sepsis	WCC, platelet count
Husada LOS (Husada 2010)	Thailand	LOS	Logistic regression	Oxygen requirement, poor feeding, abnormal heart rate, abnormal temperature		WCC, pH
Iqbal (Iqbal 2024)	India	All sepsis	Supervised machine learning	Apnoea, tachycardia, bradycardia, desaturation, lethargy, septic shock, meningitis, pneumonia, Apgar score	Prematurity, vaginal delivery, low birthweight, very low birthweight, IUGR, SGA, primigravida, central catheter, peripheral catheter, end-diastolic flow, CPAP, mechanical ventilation, inotropes, corticosteroids, cardiac disease, lung disorder, respiratory distress syndrome, intraventricular haemorrhage, necrotising enterocolitis, fungal infection	WCC, RBC count, platelet count, neutrophil count, CRP, hypocalcaemia, hypoglycaemia, metabolic acidosis
Istanbul protocol (Bulbul 2020)	Turkey	Serious bacterial infection	Criteria-based without specific scoring	Fever ≥38°C, appearance, dehydration, focal signs of infection	Perinatal antibiotics, chronic disease, hospitalised longer than mother	CRP, WCC, I:T ratio, urinalysis
Kaiser Permanente EOS Calculator (Kuzniewicz 2017)	USA	EOS	Bayesian logistic regression and recursive partitioning	Clinical presentation (well appearing, equivocal, clinical illness)	EOS incidence, gestational age, duration of ROM, highest maternal intrapartum temperature, maternal GBS, intrapartum antibiotics	

Kar (Kar 2010)	India	LOS	Scoring system, univariable positive likelihood ratio	Lethargy, tachycardia, fever >37.5°C, abdominal distention, increased prefeed aspirate, chest retractions, grunting		
Matsushita (Matsushita 2022)	Brazil	All sepsis	Supervised machine learning			Haemoglobin, haematocrit, MCV, MCH, MCHC, WCC, neutrophil %, neutrophil count, neutrophil left shift %, neutrophil left shift count, eosinophil %, eosinophil count, basophil %, basophil count, lymphocyte %, lymphocyte count, monocyte %, monocyte count, platelet count, neutrophil to lymphocyte ratio, monocyte to lymphocyte ratio, platelet to lymphocyte ratio, delta neutrophil index, CRP, lymphocyte to CRP ratio
Mondal (Mondal 2012)	India	All sepsis	Scoring system, univariable preselection			CRP, micro-ESR, I:T ratio, morphological changes in neutrophils
Neal (Neal 2023)	Zimbabwe	EOS	Logistic regression	Fever >37.5°C, respiratory rate, activity, chest retractions, grunting	PROM, maternal intrapartum fever, foul smelling liquor	
NeoHoP (Lloyd 2023)	South Africa	LOS (HAI)	Logistic regression, univariable preselection	Capillary refill time, lethargy, abdominal distention	Central venous catheter	CRP
Nguyen (Nguyen 2023)	China	All sepsis	Tree augmented naive Bayes	Overall symptoms, gastrointestinal symptoms, central nervous symptoms, skin symptoms, respiratory symptoms, cardiovascular symptoms, infective symptoms, abnormal temperature symptoms, heart rate, respiratory rate, temperature, oxygen saturation, systolic blood pressure, diastolic blood pressure	Age, gender, low birthweight, prematurity, use of vasoactive agents, ICU length of stay, hospital length of stay, in-hospital mortality	WCC, neutrophil count, lymphocyte count, platelet count, partial thromboplastin time, prothrombin time, ESR, glucose, lactate, creatinine, procalcitonin, CRP, positive microbiological test

NOSEP-1 and NOSEP-NEW1 (Mahieu 2000, Mahieu 2002)	Belgium	LOS	Logistic regression, univariable preselection and stepwise selection	Fever >38.2°C	Parenteral nutrition for ≥14 days	CRP, platelet count, neutrophil fraction
Okascharoen (Okascharoen 2005)	Thailand	LOS	Cox model, univariable preselection and backward elimination	Hypotension, abnormal temperature, respiratory insufficiency	Umbilical venous catheter duration	Band cell fraction, platelet count
Perinatal Infection Risk Score (Takkar 1974)	India	All sepsis	ND	Apgar score	Prematurity, low birthweight, PROM, foul smelling liquor, unclean vaginal examination before delivery, duration of labour exceeding 24 hours	
Philadelphia protocol (Baker 1993)	USA	Serious bacterial infection	Criteria-based without specific scoring	Appearance, focal signs of infection		WCC, I:T ratio, CSF analysis, urinalysis, stool analysis, CXR
Pokhylko (Pokhylko 2020)	Ukraine	EOS	Logistic regression, stepwise selection	Apgar score	Prematurity, PROM, visual changes in placenta, history of abortion	Monocyte count
PROM-Scoring (Afjeiee 2008)	Iran	All sepsis	Scoring system	Apgar score	Prematurity, PROM, foetal tachycardia, chorioamnionitis, low birthweight, sex	
Pukhtinskaya (Pukhtinskaya 2021)	Russia	EOS	Decision tree			CD95, nitric oxide, CD34, CD69, lymphocytes with expression AnnexinV-FITC+PI
Rochester protocol (Powell 1990)	USA	Serious bacterial infection	Criteria-based without specific scoring	Fever ≥38°C, appearance, focal signs of infection	Prematurity, perinatal antibiotics, chronic disease, hospitalised longer than mother	WCC, immature PMN count, urinalysis, stool analysis
Rosenberg (Rosenberg 2010)	Bangladesh	LOS	Logistic regression, univariable preselection	Pallor, apnoea, lethargy, jaundice, hepatomegaly		
Selimovic (Selimovic 2010)	Bosnia & Herzegovina	EOS	Logistic regression, univariable preselection			WCC, I:T ratio, I:M ratio, CRP
Septic screen (generic)	Various	All sepsis	NA			CRP, micro-ESR, WCC, neutrophil count, I:T ratio, platelet count

Shuai (Shuai 2022)	China	LOS	Logistic regression, univariable preselection		Dopamine use, PROM, albumin use, maternal age, gender, peripherally inserted central catheter, gestational age, antibiotic use, season of delivery, asphyxia, prenatal glucocorticoids, umbilical venous catheter, birth weight	
Singh (Singh 2003)	India	LOS	Scoring system, univariable positive likelihood ratio	Lethargy, tachycardia, fever >37.5°C, abdominal distention, increased prefeed aspirate, chest retractions, grunting		
STOPS tool (James 2021)	India	All sepsis	Scoring system	Fever >37.5°C, hypothermia <35.5°C, tachycardia, bradycardia, tachypnoea, oxygen requirement, respiratory distress, poor feeding, hypoglycaemia, lethargy, irritability, weak cry, spontaneous movements, seizures, temperature of hands and feet, increased ventilatory requirements, apnoea, capillary refill time, arterial hypotension, skin colour		Procalcitonin, glucose
Weber (Weber 2003)	Ethiopia, the Gambia, Papua New Guinea & the Philippines	All sepsis, meningitis, pneumonia, or hypoxaemia	Logistic regression, univariable preselection	Fever >38°C, reduced feeding, no spontaneous movement, drowsy or unconscious, history of feeding problems, history of change in activity, agitated, capillary refill time, chest wall indrawing, respiratory rate, grunting, cyanosis, seizures, bulging fontanelle		
Wu (Wu 2024)	China	All sepsis	Nomogram from logistic regression, univariable preselection		Maternal age, gestational diabetes, forceps delivery, umbilical cord winding, gender	

Yadav (Yadav 2023)	India	All sepsis	Scoring system			WCC, I:T ratio, neutrophil count, CRP, platelet count, mean platelet volume, platelet distribution width
Yin (Yin 2022)	China	Invasive bacterial infection	Decision tree from logistic regression, stepwise selection	Ill appearance, abnormal neurological signs	Age at admission	WCC, procalcitonin, CRP, neutrophil %
Zhang (Zhang 2023)	China	EOS	Logistic regression, backward elimination	Vomiting, cough	Age, intra-amniotic infection	

CD = cluster of differentiation; CPAP = continuous positive airway pressure; CRP = C-reactive protein; CSF = cerebrospinal fluid; CXR = chest x-ray; EOS = early-onset sepsis; ESR = erythrocyte sedimentation rate; GBS = Group B streptococcus; HAI = healthcare-associated infection; ICU = intensive care unit; I:M ratio = immature to mature neutrophil ratio; I:T ratio = immature to total neutrophil ratio; IUGR = intrauterine growth restriction; LOS = late-onset sepsis; MCH = mean cell haemoglobin; MCHC = mean cell haemoglobin concentration; MCV = mean cell volume; NA = not applicable; ND = no data; PMN = polymorphonuclear neutrophil; PROM = premature rupture of membranes; RBC = red blood cell; ROC = receiver operating characteristic; ROM = rupture of membranes; SGA = small for gestational age; USA = United States of America; UTI = urinary tract infection; WCC = white cell count.

Supplemental Table 3. Summary of geographical and economic distribution of included studies.

**WHO region**	**Country**	**Income classification**	***n* studies**
African Region (AFR)	Ethiopia	LIC	1
	South Africa	UMIC	2
	The Gambia	LIC	1
	Zimbabwe	LMIC	1
Eastern Mediterranean Region (EMR)	Egypt	LMIC	4
	Iran	LMIC	2
	Jordan	LMIC	1
	Pakistan	LMIC	1
European Region (EUR)	Bosnia and Herzegovina	UMIC	1
	Russia	UMIC	1
	Turkey	UMIC	2
	Ukraine	LMIC	1
Region of the Americas (AMR)	Brazil	UMIC	3
	Mexico	UMIC	2
	Paraguay	UMIC	1
South-East Asian Region (SEAR)	Bangladesh	LMIC	2
	India	LMIC	37
	Indonesia	UMIC	7
	Thailand	UMIC	2
Western Pacific Region (WPR)	China	UMIC	9
	Papua New Guinea	LMIC	1
	Philippines	LMIC	3

One study conducted in multiple countries across multiple WHO regions and income classifications. LIC = low-income country; LMIC = lower middle-income country; UMIC = upper middle-income country; WHO = World Health Organization.

Supplemental Table 4. Included studies by World Health Organization (WHO) region.

**WHO region**	***n* studies**
African Region (AFR)	4
Eastern Mediterranean Region (EMR)	8
European Region (EUR)	5
Region of the Americas (AMR)	6
South-East Asian Region (SEAR)	48
Western Pacific Region (WPR)	12

One study conducted in multiple WHO regions.

Supplemental Table 5. Included studies by World Bank 2020 income classification.

**Income classification**	***n* studies**
Lower income	1
Lower middle-income	52
Upper middle-income	30

One study conducted in countries from multiple income classifications.

Supplemental Table 6. Predictors in included models.

**Predictor**	***n* models**
Clinical features	
Abdominal distension	4
Abnormal heart rate	8
Abnormal temperature	3
Age at admission	3
Albumin use	1
Altered behaviour, altered consciousness or lethargy	9
Apgar-1	3
Apgar-5	3
Apnoea	2
Asphyxia	1
Birthweight	7
Bulging fontanelle	1
Cardiovascular symptoms	1
Catheter	1
Cervicovaginitis	1
Chorioamnionitis	4
Central nervous system symptoms	1
Colour or pallor	2
Continuous positive airway pressure	1
Cough	1
Dehydration	1
Diastolic blood pressure	1
End-diastolic flow	1
Equivocal appearance	1
Evidence of focal infection	1
Feed intolerance or increased aspirates	2
Feeding difficulty	4

Fetal or neonatal morbidity	4
Foul liquor	3
Fungal infection	1
Gastrointestinal symptoms	1
Gestational age	11
Hospital length of stay	1
Hypotension	3
Hypothermia	2
Hypoxia or oxygen saturation	3
ICU length of stay	1
Ill appearance	3
In-hospital mortality	1
Increased oxygen or ventilation requirement	4
Infective symptoms	1
Inotropes or dopamine or vasoactive drug use	3
Intrapartum antibiotics	1
Intrauterine growth restriction	1
Intubated or mechanical ventilation	3
Labour >24h	1
Length of stay pre-sepsis	1
Local incidence of sepsis	1
Maternal age	3
Maternal Group B streptococcus	1
Maternal history of abortion	1
Maternal morbidity	2
Maternal parity or gravidity	1
Maternal temperature or fever	2
Neonatal fever	13
Nitrous oxide	1
Perfusion or capillary refill time	4

Premature rupture of membrane	1
Prenatal glucocorticoid use	2
Previous hospitalisation or illness	2
Previously healthy	3
Received antibiotics	4
Received recent immunisation	1
Respiratory insufficiency or distress	7
Respiratory rate	4
Respiratory symptoms	1
Rupture of membranes duration	6
Season	1
Seizures	1
Sex	5
Signs of encephalopathy or abnormal neurological examination	1
Skin symptoms	1
Small for gestational age	1
Systolic blood pressure	1
Total parenteral nutrition	2
Type of birth	2
Urinary tract infection	1
Umbilical cord winding	1
Umbilical or central venous catheter	6
Unclean vaginal examination	1
Vomiting	1
Well appearance	5
Laboratory parameters	
Calcium	1
CD34	1
CD69	1
CD95	1

Cell volume	1
Chest x-ray	4
Conductivity for internal composition of cell	1
Creatinine	1
CRP	16
CSF white cell count	4
Degenerative changes in polymorphonuclear cells	2
Delta neutrophil index	1
Eosinophil count or percentage	1
Gastric acid cytology	1
Glucose	3
Haemoglobin	1
Haematocrit	1
I:M ratio	2
I:T ratio	9
IL-6	2
IL-27	1
Immature polymorphonuclear cell count or percentage, band cell count or percentage, or neutrophil bandaemia	4
Lactate	1
Light scatter for cytoplasmic granularity and nuclear structure	1
Lower median angle light scatter	1
Lymphocyte count or percentage	2
Lymphocyte:CRP ratio	1
Lymphocytes with expression AnnexinV-FITC+PI	1
Mean cell haemoglobin	1
Mean cell haemoglobin concentration	1
Mean cell volume	1
Mean neutrophil volume	1
Mean platelet volume	1

Micro ESR or ESR	3
Monocyte count or percentage	2
Monocyte:lymphocyte ratio	1
Neutrophil count or percentage	11
Neutrophil:lymphocyte ratio	1
Nitric oxide	1
Partial thromboplastin time	1
pH	2
Platelet count	15
Platelet distribution width	1
Platelet:lymphocyte ratio	1
Positive microbiological test	1
Procalcitonin	4
Prothrombin time	1
Red blood cell count	1
Stool white cell count	3
Thyroid function	1
Total polymorphonuclear count	1
Urine white cell count	4
Visual placental changes	1
White cell count	17
