# Supplementary material for: Clinical prediction models to diagnose neonatal sepsis in low-income and middle-income countries: a scoping review
Source: BMJ Glob Health. 2025 Apr 9;10(4):e017582. doi: 10.1136/bmjgh-2024-017582 (PMC12182008; doi:10.1136/bmjgh-2024-017582)
Supplement: online supplemental material 1 [file bmjgh-10-4-s005.docx]

**AUTHOR REFLEXIVITY STATEMENT**

**Has the research team engaged constructively with the reflexivity statement?**

This reflexivity statement has been jointly composed by all authors.

**Have the research partners co-developed the research study?**

GC is a consultant neonatologist and lecturer at the University of Zimbabwe, and DM is a specialist paediatrician in Zimbabwe. Both contributed to the development of our review protocol and played an integral part in the conduct, reporting and interpretation of this review.

**Does the study address priority research questions for the LMIC partner(s)?**

Neonatal sepsis kills many neonates globally, with most deaths occurring in low-income and middle-income countries. Over 60 countries need to improve their current progress to meet the Sustainable

Development Goals target to reduce neonatal mortality to at least as low as 12 per 1000 live births, two thirds of which are in sub-Saharan Africa. Therefore, addressing preventable and treatable causes of neonatal mortality, such as neonatal sepsis, is a global priority.

**Is there a LMIC partner who is the first or last author?**

Yes, GC is the joint last author of this study.

**How have LMIC early career researchers been incorporated as authors?**

GC is an early career researcher and is joint last author of this study. Early career researchers continue to play a crucial role in the broader objectives of our research group and are listed as authors on other publications.

**How are data shared with LMIC partners to address research needs?**

All members of our research partnership have access to data collected and generated by our research group.

**Is there open access funding to improve publication dissemination?**

Yes, this study will be published as an open access article.
